# Identification of butenolide regulatory system controlling secondary metabolism in Streptomyces albus J1074

**DOI:** 10.1038/s41598-017-10316-y

**Published:** 2017-08-29

**Authors:** Yousra Ahmed, Yuriy Rebets, Bogdan Tokovenko, Elke Brötz, Andriy Luzhetskyy

**Affiliations:** 1 Helmholtz-Institute for Pharmaceutical Research Saarland, Actinobacteria Metabolic Engineering Group, Building E8.1, 66123 Saarbrücken, Germany; 2 Universität des Saarlandes, Pharmazeutische Biotechnologie, Building C2.3, 66123 Saarbrücken, Germany; 3 Present Address: BASF SE, GBW/H -67056, Ludwigshafen am Rhein, Germany; 4 Weilburger Coatings GmbH, Ahäuser Weg 12-22, 35781 Weilburg/Lahn, Germany

## Abstract

A large majority of genome-encrypted chemical diversity in actinobacteria remains to be discovered, which is related to the low level of secondary metabolism genes expression. Here, we report the application of a reporter-guided screening strategy to activate cryptic polycyclic tetramate macrolactam gene clusters in *Streptomyces albus* J1074. The analysis of the *S. albus* transcriptome revealed an overall low level of secondary metabolism genes transcription. Combined with transposon mutagenesis, reporter-guided screening resulted in the selection of two *S. albus* strains with altered secondary metabolites production. Transposon insertion in the most prominent strain, *S. albus* ATGSal2P2::TN14, was mapped to the *XNR_3174* gene encoding an unclassified transcriptional regulator. The mutant strain was found to produce the avenolide-like compound butenolide 4. The deletion of the gene encoding a putative acyl-CoA oxidase, an orthologue of the *Streptomyces avermitilis* avenolide biosynthesis enzyme, in the *S. albus XNR_3174* mutant caused silencing of secondary metabolism. The homologues of *XNR_3174* and the butenolide biosynthesis genes were found in the genomes of multiple *Streptomyces* species. This result leads us to believe that the discovered regulatory elements comprise a new condition-dependent system that controls secondary metabolism in actinobacteria and can be manipulated to activate cryptic biosynthetic pathways.

## Introduction

The genomics era in actinobacteria research has led to rapid changes in our understanding of secondary metabolite diversity^1, 2^. A great surprise was that only a few compounds are produced under laboratory conditions by the actinobacteria strains harboring 30–40 secondary metabolite gene clusters in their genomes^3–5^. The majority of these genes are considered silent or expressed at a level insufficient for the detection of corresponding compounds. The strains (such as S*. coelicolor, S. lividans*, and *S. avermitilis*) that have been used for decades as model actinomycetes with well-studied metabolic profiles were found to produce new, unknown secondary metabolites^6, 7^. The extensive genomics-based re-thinking of the *S. coelicolor* secondary metabolome increased the number of compounds isolated from this strain from 4 to 17^8^. A simple calculation makes it obvious that a large majority of the chemical potential of actinobacteria remains unexplored, opening opportunities for the discovery of natural products with new chemical scaffolds and biological activities^2^. The development of tools and strategies for efficient genome mining for secondary metabolism genes and their activation is now the primary goal in actinobacteria genetics^9^.

Several approaches have been used to activate cryptic gene clusters in actinobacteria (for reviews see refs 9, 10). Both “unselective”, based on introducing global changes into intracellular processes, and “selective”, manipulating a specific cluster of interest, strategies have been intensively developed and applied. “Selective” approaches include engineering the transcriptional and translational processes within a cluster of interest^11–13^, overexpression of pathway-specific regulatory genes^14^, refactoring of the pathway in a plug-and-play manner^15, 16^ and heterologous expression^17^. These methods require the development of tools for manipulating large DNA fragments, efficient heterologous hosts and well-characterized libraries of gene expression elements^7, 18^. Changes in media composition^19, 20^, ribosomal and metabolic engineering^21, 22^ and manipulating pleiotropic regulatory genes^23^ often induces global changes in the secondary metabolites profile of a producing strain. A necessary prerequisite for success when using “unselective” approaches is the availability of an efficient screening platform to recognize and select colonies with a desired phenotype.

*Streptomyces albus* J1074, the derivative of the *S. albus* strain G^24^, is one of the most widely used hosts for the heterologous expression of antibiotic biosynthesis genes^25^. The great advantage of this strain is an absence of the production of endogenous secondary metabolites. Sequencing of the *S. albus* J1074 genome led to the identification via various annotations of 22 to 26 gene clusters^11, 26^. Recently^11^, a successful application of “selective” approaches to activate several secondary metabolism pathways in *S. albus* resulted in the accumulation of indigoidine, alteramides, antimycins and candicidins by recombinant strains.

Despite the obvious success of both strategies in activating cryptic antibiotic biosynthesis pathways in actinobacteria, we still lack an answer to one of the major questions raised by the discovery of the cryptic secondary metabolome: why are these genes silent? The current approaches do not provide insight into the regulatory processes silencing secondary metabolism. A reporter-guided selection of mutants with activated metabolic pathways of interest might be a tool to answer this question. This approach is based on a simple assumption that the yield of the final metabolite directly correlates with the expression level of the corresponding biosynthetic gene cluster. This strategy was successfully applied in the selection of *Aspergillus terreus* strains with increased titers of lovastatin^27^ and was later adapted to screen for *S. clavuligerus* mutants with improved clavulanic acid production^28^. Recently, this approach was used to activate jadomycin (*jad*) and gaudimycin (*pga*) biosynthesis pathways in *S. venezuelae* and *Streptomyces* sp. PGA64, respectively^29^. Here, we report the further development and exploitation of this technique by combining it with transposon mutagenesis in *S. albus* J1074. This method enables fast and simple identification and characterization of the DNA locus responsible for the activation and production of the desired metabolite, providing insights into the regulatory processes silencing the secondary metabolism in actinobacteria.

## Results

### Secondary metabolism gene clusters in *S. albus* are poorly transcribed under laboratory conditions

**Figure 1:**
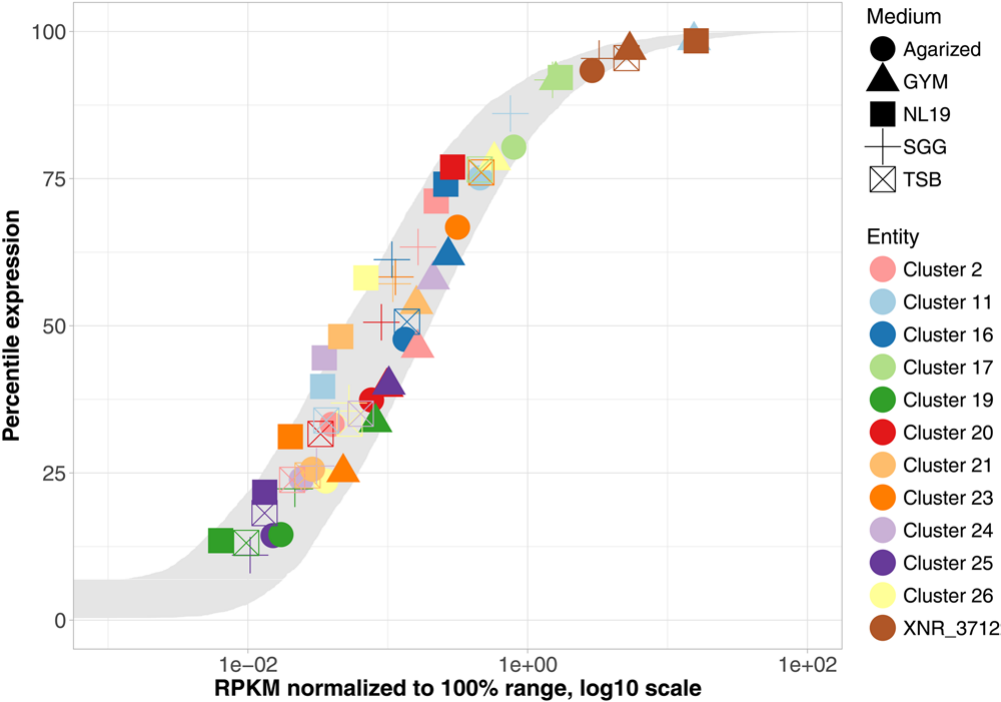
The gray ribbon shows the range of percentile-logRPKM curves for all genes within the *S. albus* J1074 genome, grown in all 7 media (for any given percentile, the ribbon covers all observed logRPKM values, including minima and maxima). Subsets of 5 media (shape-coded) and 11 gene clusters (color-coded) are also shown, with additional horizontal and vertical jitter to alleviate data-point overlaps. The *rpoB* (*XNR_3712*; brown) gene expression is shown for reference.

A manual correction of the AntiSMASH 2.0 outcome of *S. albus* J1074 genome analysis resulted in a list of 26 secondary metabolite biosynthetic gene clusters (BGCs) by removing two regions (*XNR_0391-0405* and *XNR_2192*) and adding the recently identified paulomycin BGC (*XNR_0573-0610*)^11^. To evaluate the transcriptional activity of the identified BGCs, we aggregated RNAseq datasets from several experiments in which *S. albus* J1074 was grown in various liquid media and MS agar medium^13, 26, 30^ (Fig. 1, Table 3S). The transcriptome data from these experiments were normalized by rating the level of individual gene expression in percentile from the highest expressed gene. This conversion was applied to compare data from various experiments and sequencing providers. Although this process loses most gene expression information (absolute values and fold-changes within samples), it maintains the relative order of ranked gene expression, which is sufficient for basic qualitative comparisons. The highest expressed gene for all conditions was *XNR_3521*, which encodes the putative excisionase/DNA-binding protein with RPKM values ranging from 8,641 to 15,730 (Fig. 1, Table 3S). These values were taken as 100%.

We found that *S. albus* J1074 secondary metabolism BGCs are transcriptionally active over a wide range of percentile values (Fig. 1, Table 3S). However, in the majority of BGC-medium combinations, the percentile expression was below 60–70%, with the 50 percentile ranking corresponding to values as low as 6–28 RPKM in various experiments. For comparison, the observed percentile expression across all media for *rpoB* was 93.4–98.2% (448–1,504 RPKM) and for *rps12* was 98.5–99.7% (2,683–9,949 RPKM).

The only highly expressed BGC (number 17) under tested conditions was predicted to be responsible for the biosynthesis of ectoine. Its rank-expression was 76.5–92.3% across all media (70–310 RPKM). Surprisingly, a high level of transcription was detected for the putative type I lantipeptide gene cluster (BGC 11) when the strain was grown in GYM medium (ranked at 98%, 2218 RPKM) (Table 3S). The above-described BGCs are exceptions; the majority of genes involved in secondary metabolism in the *S. albus* J1074 genome are poorly transcribed under the examined conditions at levels that appear insufficient for producing detectable amounts of metabolites.

### Reporter-guided selection of mutants with activation of polycyclic tetramate macrolactam (PTM) biosynthesis gene cluster

**Figure 2:**
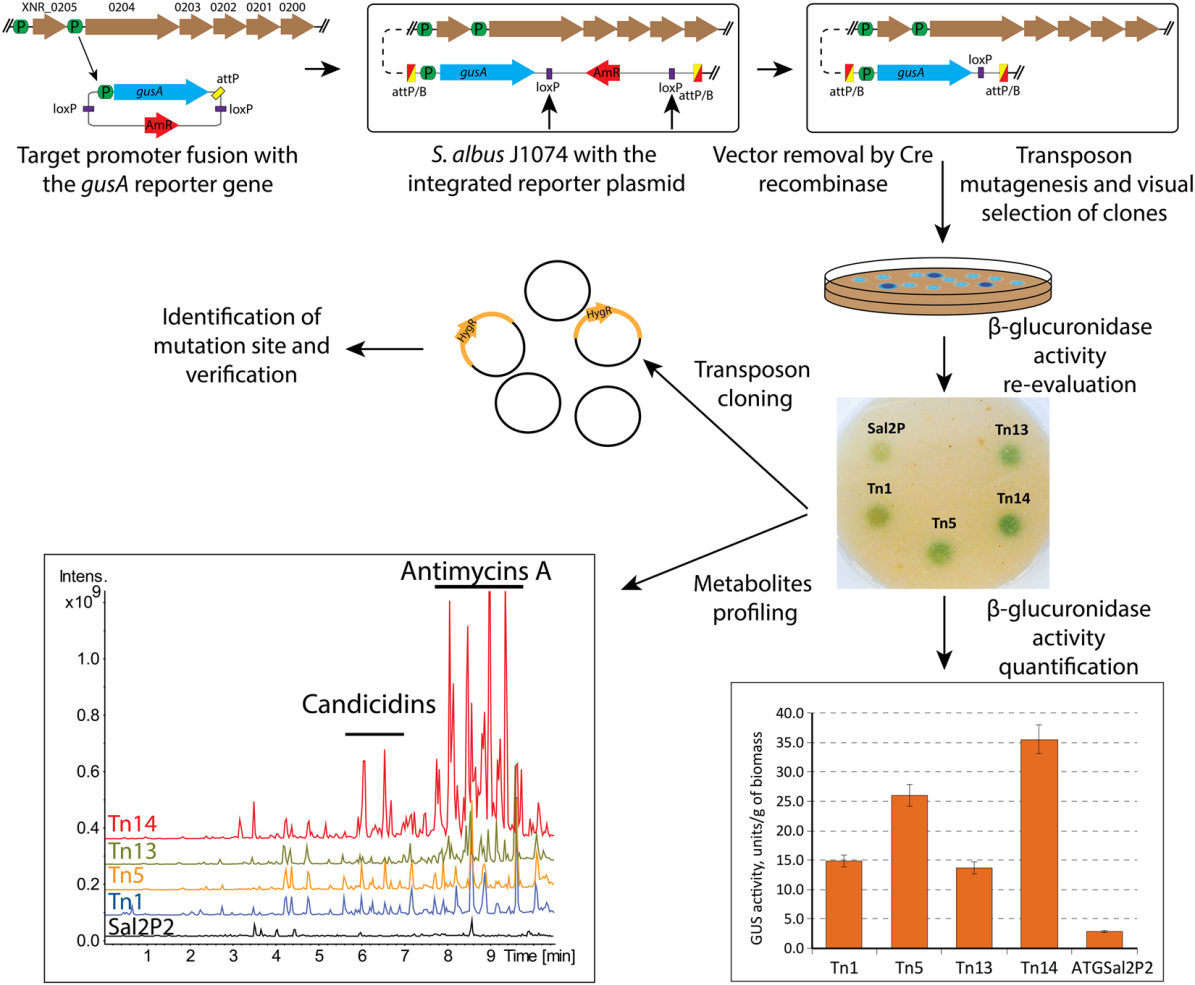
Schematic illustration of the workflow.

The gene cluster BGC 2 encodes a hybrid type I PKS-NRPS. Its activation, by the insertion of a strong promoter, led to accumulation of the 6-*epi*-alteramides A and B^11^. Cluster 2 is among the most poorly transcribed in the *S. albus* genome, ranked at the 32–70 percentile with average read counts of 6 to 23 RPKM in the various media (Fig. 1, Table 3S). The transcriptome data clearly showed that BGC 2 is organized into two transcriptional units. The ORFs *XNR_0204, 0203, 0202* and 0201 encode a hybrid type I PKS-NRPS, two oxidoreductases and a zinc-dependent alcohol dehydrogenase (cyclase) to form an operon, with the predicted transcription start site at 29 bp upstream from the start codon of *XNR_0204* (Fig. 2S). From the available RNAseq data, we cannot tell whether *XNR_0200*, which encodes the putative cytochrome P450, is also transcribed as part of this operon. *XNR_0205*, coding for putative hydroxylase/desaturase, is transcribed as monocistronic mRNA from the promoter with the (+1) position mapped 31 bp upstream of the start codon (data not shown).

We cloned a 184-bp DNA fragment, including a promoter, RBS and the first 18 bp of the coding region of the *XNR_0204* gene into the plasmid carrying the ATG allele of the *gusA* reporter gene (Figs 2, 1S, 2S)^31^. The transcriptional fusion construct facilitated rapid and simple evaluation of *XNR_0204* promoter activity, enabling visual selection of clones with increased transcription. The fusion construct was introduced into the *S. albus* J1074 strain, and the β-glucuronidase activity was tested. The recombinant strain *S. albus* ATGSal2P2 displayed a very low level of β-glucuronidase activity *in vivo* (Fig. 2). The *himar1* transposon system^32^ was applied to generate a mutant library of *S. albus* ATGSal2P2, which was further visually screened for clones with increased β-glucuronidase activity. As a result, nine clones were pre-selected from approximately 20,000 colonies.

**Figure 3:**
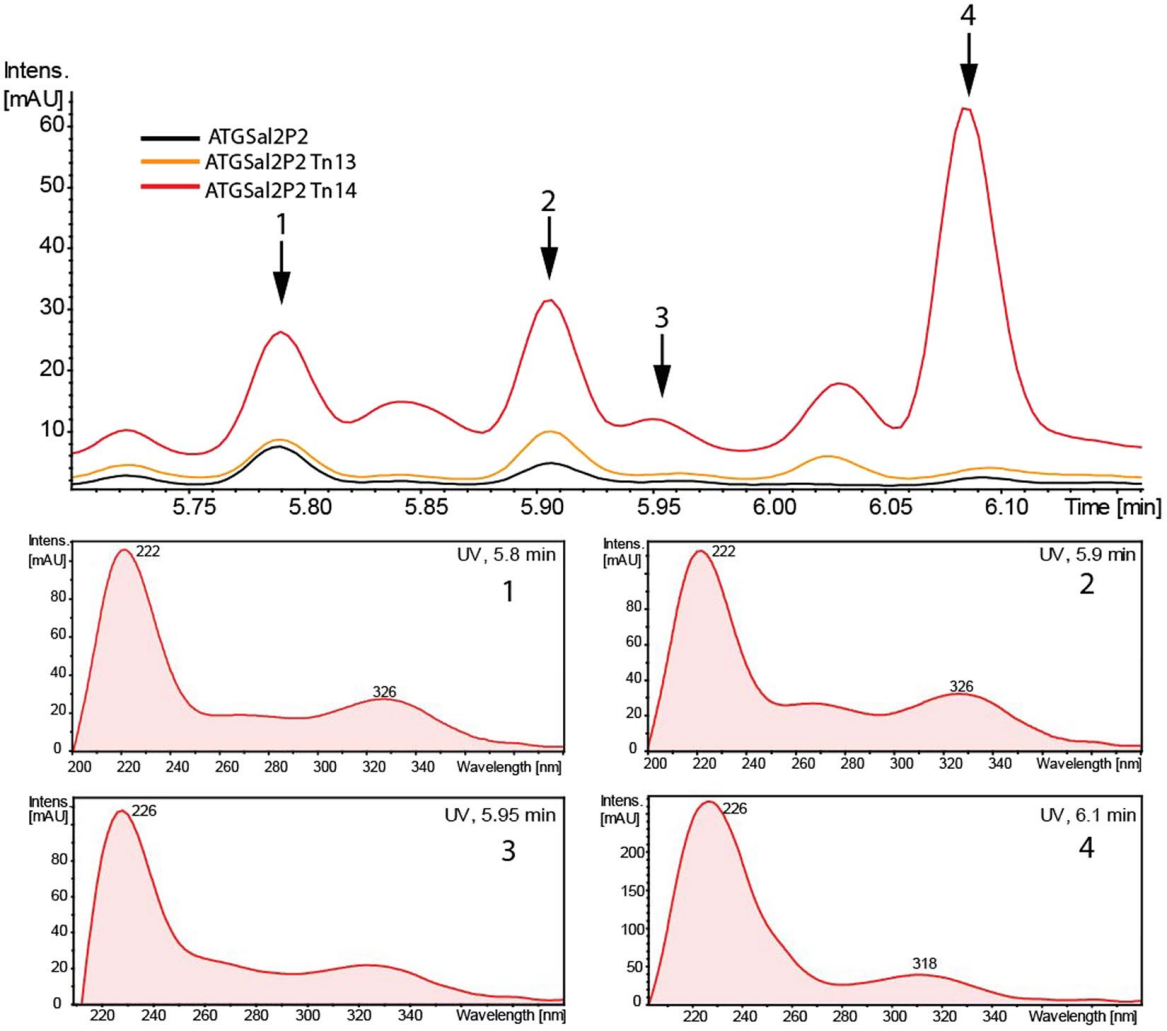
LC-MS-based identification of PTMs produced by *S. albus* ATGSal2P2::Tn13 and *S. albus* ATGSal2P2::Tn14 strains. Samples were separated with a 10 min gradient (see Materials and Methods, section 2.8).

**Figure 4:**
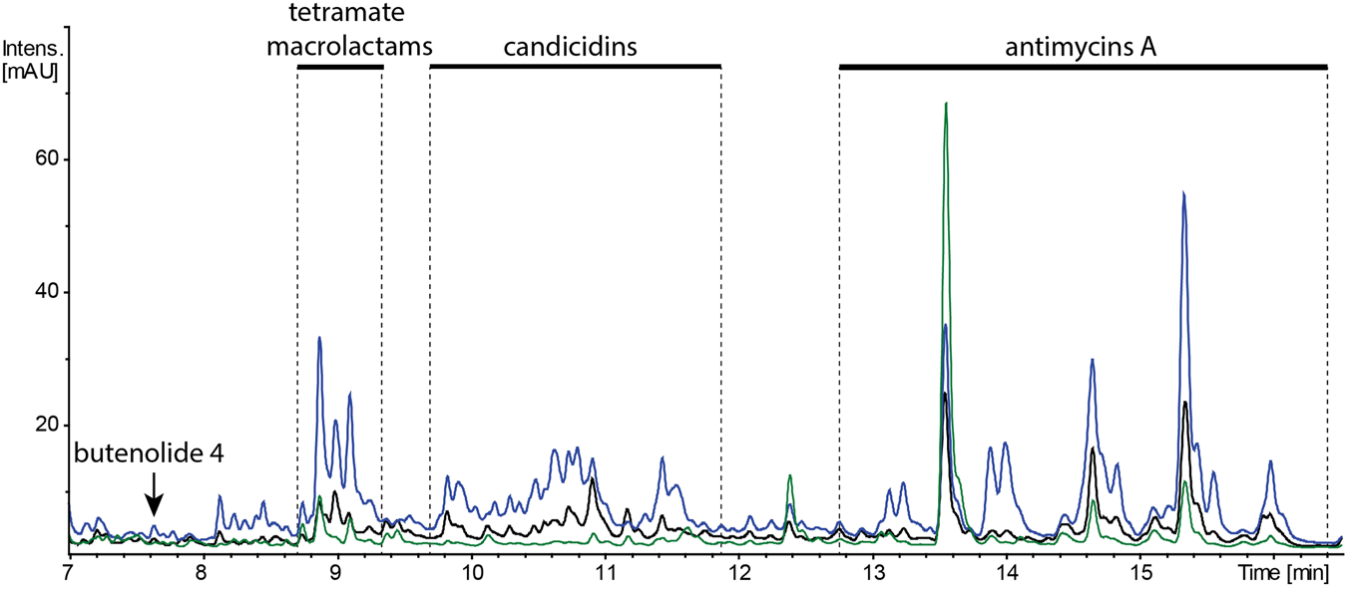
LC-MS chromatogram (at 320 nm) of extracts of *S. albus* J1074 (black), *S. albus* Δ3174 (blue) and *S. albus* Δ3174Δ2339 (green). Groups of identified compounds are highlighted. Metabolites were separated with a 20 min gradient (see Materials and Methods, section 2.8).

The selected strains were cultivated in NL19 medium, a liquid analog of the MS medium used for screening, and were analyzed for β-glucuronidase activity in cell-free lysate. Only four of nine mutants exhibited increased *gusA* expression (Fig. 2). Among these, *S. albus* ATGSal2P2::Tn14 showed the highest level of reporter activity. The strains were cultivated in several media and the produced metabolites were analyzed by LC-MS. *S. albus* ATGSal2P2::Tn13 and ATGSal2P2::Tn14 were found to produce candicidins and antimycins when grown in R5A and NL19 media, with the latter strain ATGSal2P2::Tn14 showing a significant increase in the accumulation of these compounds (Figs 2, 3 S, 4S). The metabolic profiles of two other mutants were not affected when compared to the parental strain. The rate of false positive clones was approximately 50%, as expected for a single reported gene selection procedure^29^.

**Figure 5:**
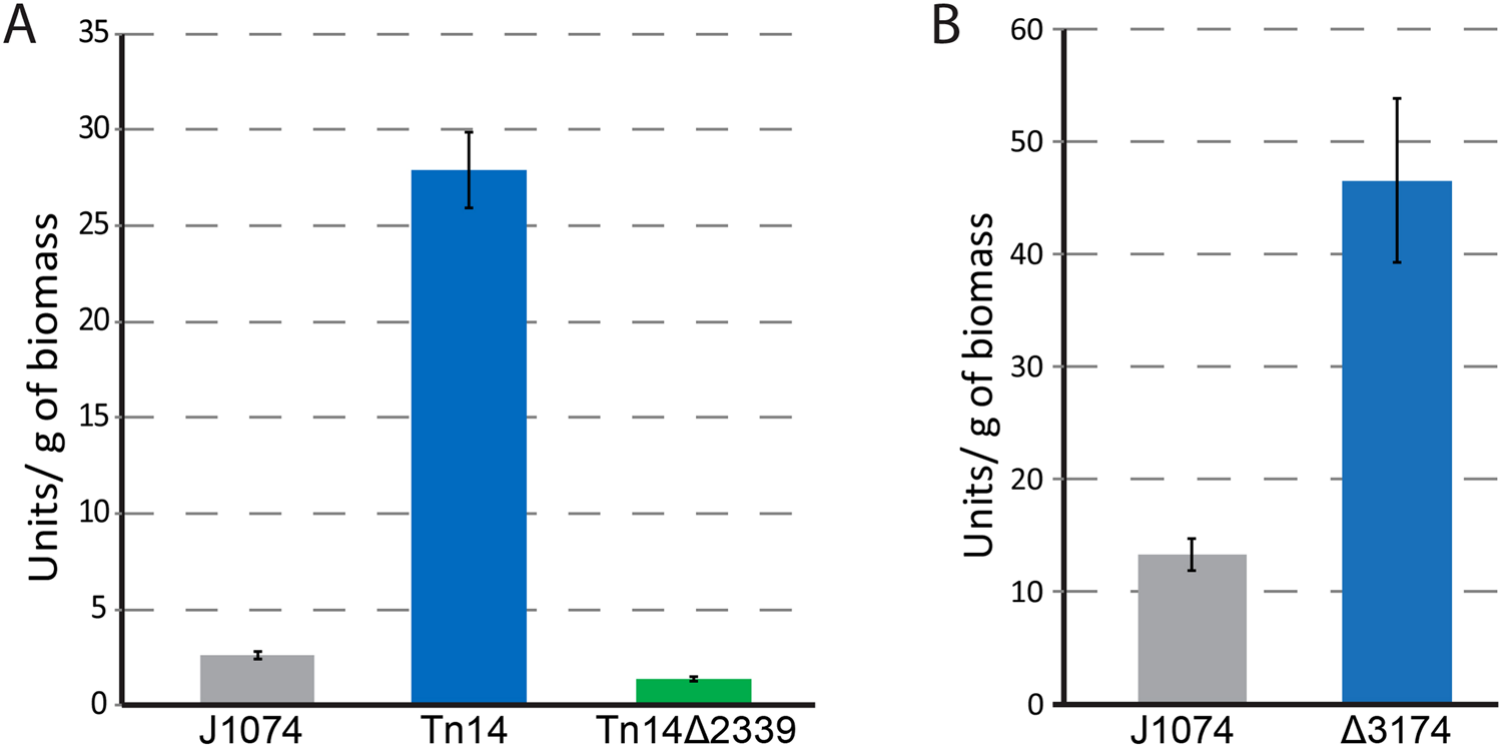
(**A**) β-glucuronidase activity of cell-free extracts of *S. albus* J1074 (black), *S. albus* ATGSal2p2::Tn14 and *S. albus* ATGSal2p2::Tn14Δ2339 (green) resulting from the *gusA* reporter expressed with the promoter of *XNR_0204* gene. (**B)** β-glucuronidase activity of cell-free extracts of *S. albus* J1074 (black) and *S. albus* Δ3174 (blue) resulted from the *gusA* reporter expressed with the promoter of *XNR_2339* gene.

**Figure 6:**
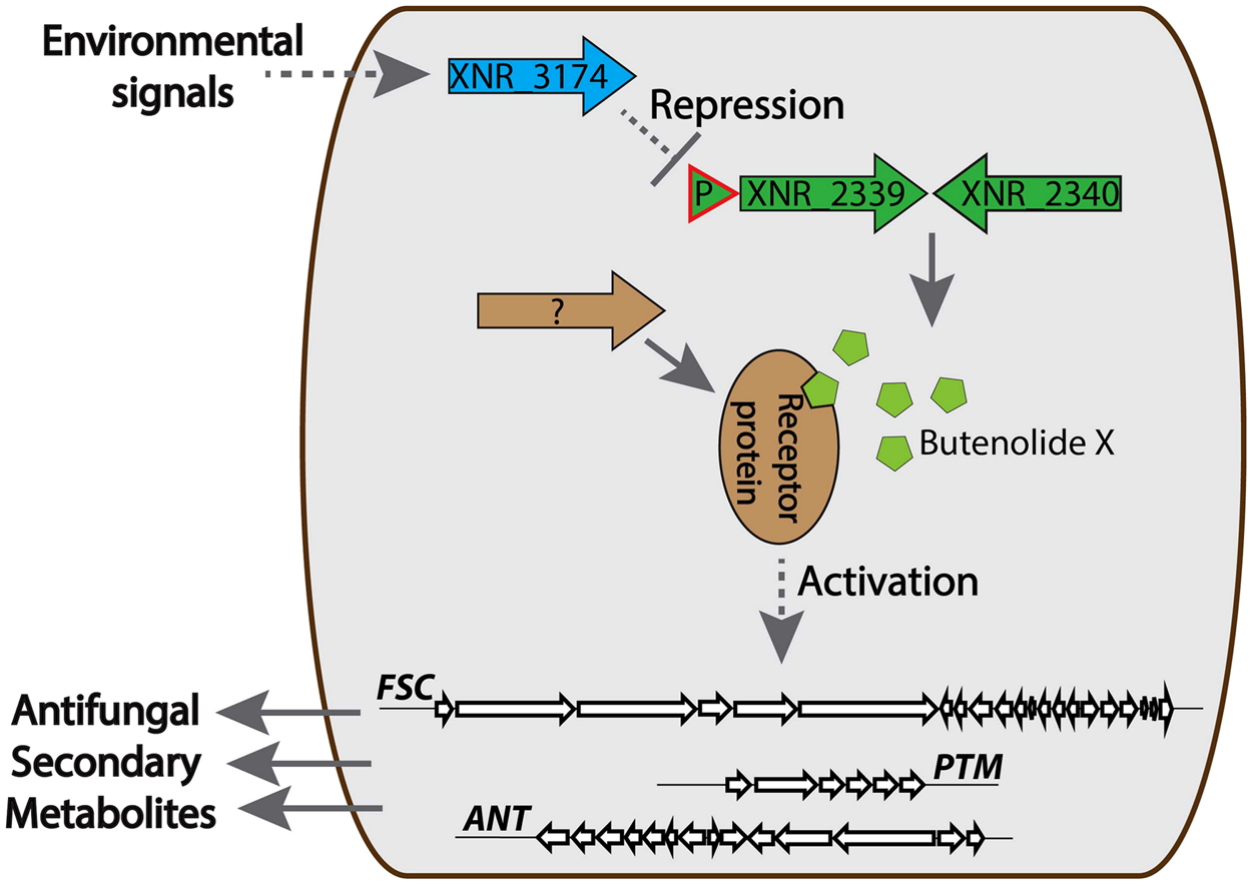
Proposed scheme of regulation of antifungal secondary metabolites (PTMs, candicidins and antimycins) biosynthesis in *S. albus* J1074.

Two compounds with absorption spectra typical of PTMs^11^ can be found in the extracts of *S. albus* ATGSal2P2::Tn13 and *S. albus* ATGSal2P2::Tn14, as well as in the parental strain (compounds **1** and **2**; retention time of 5.8 and 5.9 minutes, respectively) (Fig. 3). *S. albus* ATGSal2P2::Tn13 showed a minor increase in the production of compound **2**. The *S. albus* ATGSal2P2::Tn14 strain not only accumulates more of compounds **1** and **2** but also produces several other compounds with similar spectral characteristics (Figs 3, 5S). Due to the low yield of these metabolites and their partial overlap with candicidins during separation, we grew *S. albus* ATGSal2P2::Tn14 in 5 liters of NL19 medium and fractionated the extract by size-exclusion chromatography prior to high resolution LC-MS and MS/MS analysis. Fraction 11 was found to contain five different compounds, two of which (**1** and **2**) were originally detected in the extracts of *S. albus* ATGSal2P2 and both mutants, and three (**3**, **4**, **5**) were present only in the extract of the *S. albus* ATGSal2P2::Tn14 strain (Figs 3, 5S). While we were not able to purify the detected compounds for detailed structure elucidation, all of them have MS/MS fragmentation pattern similar to pure ikarugamycin sample with three characteristic ions, that seems to be common for entire PTMs family (Fig. 6S). Based in exact mass and fragmentation pattern we suggest that compound **2** might be alteramide A (m/z of 511.2678 [M + H]^+^; calculated m/z 511.2763 [M + H]^+^) (Figs 5 S, 6S), when ccompounds **3** with m/z 509.2562 [M + H]^+^ and **5** with m/z of 525.2552 [M + H]^+^ most probably are frontalamides B and A, respectively (calculated m/z B 509.2645 [M + H]^+^ and A 525.2594 [M + H]^+^) (Fig. 7S). Nevertheless, despite the lack of exact structures of detected compounds, we can clearly state that they belong to PTMs family and originated from the activation of BGC2.

### XNR_3174 represses biosynthesis of secondary metabolites in *S. albus* J1074

The transposon insertion loci were retrieved from the chromosome of mutant strains and sequenced (Table 4S). The transposon insertion in *S. albus* ATGSal2P2::Tn13 occurred in the *XNR_3521* gene, which encodes a putative MerR family transcription factor. This protein was found to be highly conserved among various actinobacteria, with the degree of amino acid identity ranging from 86% to 100%. In the case of *S. albus* ATGSal2P2::Tn14, the transposon was mapped to the 3′ end of the coding region of the *XNR_3174* gene. The *XNR_3174* product is annotated as a putative transcriptional regulator with unknown function^26^. This gene is transcribed as monocystronic mRNA and showed high transcriptional activity in the cultures of *S. albus* J1074 grown in NL19 or solid MS media, but not in SGG or TSB (Table 3S). Transposon insertion disrupts the *XNR_3174* gene by cutting the last 13 codons. The domain BLAST analysis showed that this region of the protein is predicted to form a helix-turn-helix DNA binding motif.

Because *S. albus* ATGSal2P2::Tn14 transposon insertion showed the most prominent phenotypic expression, the *XNR_3174* gene was deleted in the chromosome of *S. albus* J1074 to verify the mutant phenotype. The obtained strain *S. albus* Δ3174 demonstrated a similar metabolic profile to *S. albus* ATGSal2P2::Tn14, accumulating PTMs, candicidins and antimycins when grown in NL19 medium (Fig. 4). The reintroduction of *XNR_3174* on a plasmid into *S. albus* Δ3174 resulted in cessation of PTM and candicidins production and a significant decrease in antimycins accumulation (data not shown).

### Putative butenolide biosynthesis genes are involved in the regulation of secondary metabolism in *S. albus* J1074

Analysis of extracts from the *S. albus* ATGSal2P2::Tn14 and Δ3174 strains revealed one compound with *m/z* 225.1373 [M + H]^+^ that was not produced by the parental strain under the tested conditions (Figs 4, 8S). This compound was purified, and its structure was established by NMR experiments to be 4-hydroxy-10-methyl-11-oxo-dodec-2-en-1,4-olide (butenolide 4) (Table 5S, Fig. 9S) This compound, together with several other derivatives, was previously isolated from extracts of marine *Streptomyces* sp. SM8 and *S*. sp. B3497^33, 34^, and is structurally related to avenolide, the chemical trigger of secondary metabolism in *S. avermitilis* ^35^. However, we did not detect avenolide itself in the extracts of the *S. albus* ATGSal2P2::Tn14 or Δ3174 strains.

The biosynthesis of avenolide in *S. avermitilis* is proposed to be governed by two enzymes: acyl-CoA oxidase and cytochrome P450 hydroxylase^35^. Genes encoding these enzymes (*aco* and *cyp17*, respectively) are clustered with the gene for the avenolide receptor protein AvaR1. We searched the genome of *S. albus* J1074 for the *aco* and *cyp17* orthologues, excluding *avaR1* from the search. Two genes, *XNR_2339* and *XNR_2340*, encoding a putative acyl-CoA oxidase and P450 hydroxylase, were found to be the closest homologues of *aco* and *cyp17* in the *S. albus* J1074 genome (Table 6S). These genes are poorly transcribed in *S. albus* J1074 according to RNAseq data (Table 3S). Additional genome mining of the *avaR1* homologue resulted in detection of the *XNR_4681* gene as a possible hit (31% identity and 43% similarity of protein sequences).

The *XNR_2339* gene was deleted from the chromosome of *S. albus* ATGSal2P2::Tn14 and *S. albus* Δ3174. The obtained mutants lost the ability to accumulate butenolide 4 and had significantly decreased production of PTMs, candicidins and antimycins (Figs 4, 8S). At the same time, transcription from the *XNR_0204* promoter driving the expression of the PTM synthase gene was severely decreased in the double mutant compared to the *S. albus* ATGSal2P2::Tn14 strain (Fig. 5A). The genetic complementation of *S. albus* Tn14Δ2339 with native *XNR_2339* led to restoration of secondary metabolite production (data not shown). Furthermore, the overexpression of the *XNR_2339* gene from the *ermE* promoter induced secondary metabolism in *S. albus* J1074 (Fig. 10S).

We generated the transcriptional fusion of the *XNR_2339* gene promoter (a 273-bp DNA fragment including the promoter and the 98-bp coding region of *XNR_2339*) with the *gusA* reporter. The resulting construct was introduced into *S. albus* J1074 and *S. albus* Δ3174, and the β-glucuronidase activity was measured in the cell-free extract (Fig. 5B). The *XNR_2339* promoter was found to be 6- to 10-fold more active in *S. albus* Δ3174 than in the wild-type strain, suggesting that the *XNR_3174* transcriptional factor represses the expression of the *XNR_2339* and *XNR_2340* genes responsible for production of the avenolide-like chemical trigger, rather than directly influencing secondary metabolites biosynthesis genes expression in *S. albus*. The deletion of *XNR_4681*, which encodes a putative butenolide receptor protein, did not influence production of secondary metabolites by *S. albus* J1074 (data not shown).

### XNR_3174 influence the production of heterologous secondary metabolites in *S. albus*

Four gene clusters encoding biosynthesis pathways for pamamycin, aranciamycin, griseorhodin and tunicamycin were introduced into *S. albus* J1074 and Δ3174. Production of corresponding compounds was examined in three different media (NL19, SM17 and SG for aranciamycin, griseorhodin and tunicamycin or SGG for pamamycin). Metabolites of polyketide origin (pamamycin, aranciamycin and griseorhodin) were overproduced in the mutant strain when cultivated in NL19 and SG (or SGG for pamamycin) media (Fig. 11S). In average, up to 5 fold increase in griseorhodin, 14 fold in pamamycin and 5 fold in aranciamycin accumulation was observed. The observed phenomenon was clearly media dependent, since there were no changes in production of these metabolites by SM17 grown cultures of *S. albus* Δ3174. In the same time, biosynthesis of tunicamycin and moenomycin (data not shown) was not affected by the deletion of *XNR_3174* at any tested conditions. Therefore, *XNR_3174* influence the expression of heterologous polyketide biosynthesis gene clusters in *S. albus*. This correlates with the observed increase in production of intrinsic PKS and hybrid PKS-NRPS products in *S. albus* Δ3174.

### Butenolides are an alternative hormone system in actinobacteria dedicated to secondary metabolism

To estimate the distribution of the butenolide regulatory system among different actinobacteria, we performed a BLAST search for homologs of the Aco and Cyp17 proteins from *S. avermitilis*, XNR_2339, XNR_2340 and XNR_3174 from *S. albus* J1074 and the A-factor synthase AfsA from *S. griseus* within the actinobacteria genomes available from public databases (Table 6S). As many genomes are only available as draft sequences, the proximity of the *aco* and *cyp17* orthologues was excluded from the search criteria. The putative butenolide biosynthesis genes were only found in the genus *Streptomyces* (Table 6S). Among the 415 streptomycetes genomes searched, putative butenolide biosynthesis enzymes were present in 74 strains (aa identity threshold set to 50%). Among these, 19 strains can be categorized into a provisional *S. albus* sub-group (Table 6S, Fig. 12S). These strains not only showed a high degree of conservation of XNR_2339, XNR_2340 and XNR_3174 proteins but also carried the putative antimycin, candicidin and PTM biosynthesis gene clusters within their genomes. Several of these strains also encode putative AfsA homologues, whereas others did not. One of these strains, *S. albidoflavus NRRL B-1271*, was grown in NL19 media, and the extracted metabolites were analyzed by LC-MS. The strain was found to produce a set of candicidins and butenolide 4 (Fig. 13S). At least two other strains from the *S. albus* sub-group, *Streptomyces sp*. S4 and *Streptomyces sp*. SM8, are known to produce antimycins and candicidins^34, 36^.

## Discussion

The production of secondary metabolites in actinobacteria is controlled by environmental factors through diverse regulatory genes and networks^37^. In all cases, regulation occurs in response to internal or external signals that are often related to changes in cultivation conditions and aging of the culture. The laboratory conditions are obviously different from those that bacteria face in natural environments. These differences are believed to be reflected in the secondary metabolite repertoire of actinobacteria. Manipulation of cultivation conditions is often used to induce the production of new chemicals by seemingly well-characterized strains^19, 38^. However, the utility of this approach emphasizes the gaps in our understanding of environmental factors and corresponding intracellular regulatory processes that cause silencing of some secondary metabolism pathways while maintaining others.

The majority of secondary metabolism gene clusters discovered within actinobacteria genomes are considered silent under laboratory conditions. It is thought that silencing occurs at the transcriptional level, suggesting that activation approaches can be developed on the basis of modifying transcriptional control elements of biosynthetic genes^18^. The transcriptomic data analyzed in this work suggest that secondary metabolism genes in *S. albus* J1074 are transcriptionally active (Fig. 1, Table 3S). However, most BGCs are transcribed at a very low level that appears to be insufficient for producing detectable amounts of corresponding metabolites. In some cases, such as BGC 11, the transcription of the structural genes is sufficiently high for producing the compound, and the limitations lie in the area of metabolite detection and identification. This was clearly shown in the case of paulomycins^11^. However, efficient expression of some secondary metabolite gene clusters, such as the one for desferrioxamine, appears to be indispensable^39^.

Many approaches and strategies have been developed and successfully applied for the activation of silent secondary metabolite biosynthesis gene clusters (for a review see ref. 9). Some of these strategies (mainly “unselective” ones) involve intensive screening procedures. However, the ability to genetically manipulate actinobacteria strains outperforms the throughput of existing screening techniques. This is when the reporter-guided selection of clones with increased transcription of genes for a metabolic pathway of interest can be truly beneficial. This approach, combined with mutagenesis, was first applied to select lovastatin-overproducing *Aspergillus terreus* variants^27^ and was recently adapted to facilitate the activation of secondary metabolism in streptomycetes^29^. In both cases, conventional mutagenesis was used to generate mutant libraries, making localization of mutations difficult. Here, we combined the reporter-guided screening approach with transposon mutagenesis to awake the poorly expressed *S. albus* J1074 PTM gene cluster. The choice of *gusA* as a reporter was prompted by the simplicity of β-glucuronidase activity detection and quantification both *in vivo* and *in vitro* compared to other reporter systems for actinobacteria^7, 31^. However, the use of a single reporter resulted in a high rate of false positive clones selection (approximately 50%). The utilization of a second reporter gene, as proposed by Guo and co-authors, will decrease this number^29^. Moreover, the combination of the described approach with transposon mutagenesis enables simple and rapid identification of mutated loci, eliminating the need for genome re-sequencing^32^. The obvious bottleneck of the transposon technique, the ability to generate only inactivation mutants, can be easily overcome with a transposon carrying strong constitutive promoters at the ends of the transposable construct^40^.

Using the proposed approach, we selected four strains with increased promoter activity for the *XNR_0204* gene, encoding a putative NRPS-PKS enzyme from the PTM biosynthesis gene cluster. This cluster shares high similarity with frontalamide biosynthesis genes from *Streptomyces sp*. SPB78 (61–83% amino acid identity and 73–90% amino acid similarity among individual proteins, including the putative oxidoreductases/cyclases XNR_0203, 0202 and 0201 and hydroxylase XNR_0205)^41^. However, the attempt to activate BGC 2 resulted in the production of *6-epi*-alteramides rather than frontalamides^11^. We detected the compound with similar to alteramide A features in the extract of *S. albus* J1074 and two obtained mutants *S. albus* ATGSal2P2::Tn13 and ATGSal2P2::Tn14 (Figs 3, 5S). At the same time, the ATGSal2P2::Tn14 strain was found to produce two other PTMs with exact mass, adsorption spectra and MS/MS fragmentation pattern similar to frontalamides A and B or highly related compounds. The alteramides were previously proposed to be the shunt products of the PTMs assembly line^42^. Inactivation of the cyclase gene *ikaC* in ikaguramycin biosynthesis or OX2 or OX4 from the HSAF gene cluster (heat-stable antifungal factor, with the same cyclization pattern as frontalamides) was shown to lead to accumulation of alteramides^43, 44^. Furthermore, the ectopic expression of several PTM gene clusters resulted in accumulation of alteramides as a consequence of spontaneous cyclization of the common biosynthetic precursor^15, 45^. This result suggests that the final products of *S. albus* BGC 2 most probably are frontalamides or related PTM compounds rather than alteramides, as previously thought^11^. Notably, the properly balanced expression of the secondary metabolite biosynthesis gene clusters appears to be crucial for formation of the appropriate product. Similar observations were recently made during an attempt to express the landomycin A biosynthesis gene cluster from a strong constitutive promoter^13^. In addition to the PTMs, *S. albus* ATGSal2P2::Tn13 and ATGSal2P2::Tn14 were found to produce antimycins and candicidins.

The mutation in *S. albus* ATGSal2P2::Tn13 was mapped to the gene encoding the MerR family transcriptional regulator, whereas in *S. albus* ATGSal2P2::Tn14, transposon insertion occurred in the *XNR_3174* gene, which encodes a putative uncharacterized transcriptional factor with a typical helix-turn-helix DNA binding motif (Table 4S). Deletion of *XNR_3174* in the *S. albus* J1074 did not affect the growth rate or morphological development of the strain and only influenced secondary metabolism. Furthermore, the effect of mutation in *XNR_3174* was more evident when the strain was cultivated on MS or in NL19 media, which was used in the original screening procedure. This result suggests that the identified regulatory system is dependent on growth conditions and responds to factors present in the particular medium.

Both *S. albus* ATGSal2P2::Tn14 and of *S. albus* Δ3174 were found to produce butenolide 4. This compound structurally resembles avenolide, the chemical trigger of avermectin production in *S. avermitilis* ^35^. Butenolide 4 itself was not able to induce antibiotic production in *S. albus* J1074. However, the overexpression of the *XNR_2339* gene, encoding the orthologue of the avenolide biosynthesis acyl-CoA oxidase from the *ermE* promoter in *S. albus* J1074, led to activation of PTMs, antimycins and candicidins production. This result suggests that the activation of secondary metabolism in *S. albus* ATGSal2P2::Tn14 and *S. albus* Δ3174 strains is triggered by an as yet unidentified avenolide-like chemical messenger rather than is the direct outcome of *XNR_3174* inactivation (Fig. 6). In turn, the *XNR_3174* gene products appear to act as a repressor of this chemical messenger production because the expression of *XNR_2339* is negligible in *S. albus* J1074 and severely increased in *S. albus* ATGSal2P2::Tn14 and Δ3174 (Fig. 5). Additionally, expression of the *XNR_0204* gene strongly depends on the presence of *XNR_2339* in the chromosome of the strain. Interestingly, the discovered regulatory cascade not only controls the production of intrinsic secondary metabolites, but also influences the expression of heterologous gene clusters. This knowledge can be used to generate a better host strains for production of secondary metabolites.

Homologues of avenolide biosynthesis genes and the *XNR_3174* transcriptional regulator were found within the genomes of 74 *Streptomyces* strains (Table 6S). Among them, 19 strains can clearly be distinguished based on the presence of XNR_3174, XNR_2339 and XNR_2340 orthologues. Furthermore, almost all 19 strains carry putative gene clusters for antimycin, candicidin and PTM biosynthesis within their genomes. One strain, *Streptomyces* sp. SM8, is known to produce both antimycins and butenolides 1–3 and 4^34^. We were also able to find candicidins and butenolide 4 in the extract of *S. albidoflavus NRRL B-1271* (Fig. 13S). The high degree of conservation of the XNR_3174, XNR_2339 and XNR_2340 proteins among various streptomycetes and a clear link between these regulatory elements and several secondary metabolite biosynthesis gene clusters indicate the universality of the detected regulatory system. Furthermore, the identified regulatory mechanism controls the production of secondary metabolites with antifungal activities in response to environmental triggers. This finding may indicate the physiological and ecological significance of such a regulatory cascade for the producing strains.

## Material and Methods

### Strains, plasmids and cultures conditions

All strains and plasmids used in this work are listed in Table 1S. *E. coli* strains were grown in LB medium^46^. *S. albus* strains were grown on mannitol soy flour agar (MS agar)^47^ and in liquid TSB medium (Sigma-Aldrich, USA). NL19 (MS medium without agar), SG^48^, NL5^49^, NL111^50^, SGG^51^, SM17 (glucose 2 g/L, glycerol 40 g/L, soluble starch 2 g/L, soya flour 5 g/L, peptone (Oxoid L37) 5 g/L, yeast extract 5 g/L, NaCl 5 g/L, CaCO_3_ 2 g/L, tap water) and R5A^47^ media were used for secondary metabolites production. The following antibiotics were used when required: apramycin (50 µg/mL), kanamycin (30 µg/mL), hygromycin (50 µg/mL), thiostrepton (50 µg/mL) and phosphomycin (100 µg/mL) (Carl Roth, Germany, Sigma-Aldrich, USA).

### Isolation and manipulation of DNA

DNA extraction and manipulation, *E. coli* transformation and *E. coli*/*Streptomyces* intergeneric conjugation were performed according to standard protocols^46, 47, 52^. Dream Taq polymerase (Thermo Fisher Scientific, USA) was used to amplify DNA fragments for cloning, for PCR verification constructs and strains. DNA fragments were purified from agarose gels using the QIAquick Gel Extraction Kit (Qiagen, Germany). Plasmid and chromosomal DNA were purified with QIAprep Spin miniprep kit and DNeasy Blood and Tissue Kit (Qiagen, Germany). Restriction endonucleases and ligase were used accordingly to manufacturer recommendations (New England Biolabs, USA). Oligonucleotides used in this study are listed in Table 2S (Eurofins Genomics, Germany).

### Construction of *S. albus* ATGSal2P2 and transposon mutagenesis

Promoter of *XNR_0204* gene was amplified with the primers Sal2P2F and Sal2P2R and cloned using the CloneJET PCR Cloning Kit (Thermo Fisher Scientific, USA). The insert was sequenced and cloned by *Kpn*I-*Eco*RV into pGUS-MF-PVV vector carrying *gusA* reporter gene with ATG start codon (Fig. 1S)^31^. The resulting plasmid was introduced into *S. albus* J1074 and vector backbone was removed by expressing the Cre recombinase^53^. Obtained strain *S. albus* ATGSal2P2 was tested for β-glucuronidase activity when grown on MS and in NL19 media as described^31^. 1 mL of pre-culture was inoculated into 50 mL of NL19 media and grown for 36 h or 48 h. 2 mL of culture were taken for β-glucuronidase activity measurements in cell free extract. Data was normalized to biomass weight. Measurements were triplicated.

Transposon mutagenesis was performed as described^32^ with small modifications. Briefly, the transposon was introduced on pHTM vector, carrying *himar1* transposase gene and apramycin resistance gene within the transposable cassette and hygromycin resistance in the delivery vector. Culture was grown for 2 h in TSB medium at 30 °C and transposition was induced with 5 μg/mL of thiostrepton. After incubation at 30 °C for 4 h the temperature was raised to 39 °C for 12 or 24 h. Cells were plated on MS plates containing apramycin, incubated for 4 days at 30 °C, spores were harvested, diluted and plated out on MS plates supplemented with apramycin. Obtained colonies were transferred to TSB plates supplemented with apramycin or hygromycin to test the frequency of vector lost. The transposon library was taken for further screening if the frequency of hygromycin resistant colonies was lower than 5%. The serial dilutions of selected transposon library were plated on MS plates supplemented with 50 µg/mL of X-Gluc (X-CLUC Direct, USA), incubated for 3 days and colonies were selected by intensity and size of the blue hallo.

### Cloning and sequencing of transposon insertion loci

The genomic DNA of transposon mutants was isolated, digested with SacII, precipitated, self-ligated with T4-DNA ligase and transformed into *E. coli* TransforMax™ EC100D™ pir-116 (Epicentre, Madison, WI, USA). The plasmids were isolated, and chromosome-transposon junctions were sequenced using the primer p3-pALG-ch Sequences were mapped to the *S. albus* J1074 genome using Geneious software, version 8.1.7 (Biomatters Ltd, New Zealand).

### Recombinant BAC construction and gene deletions

Gene deletion was performed using a Red/ET recombination approach using BAC clones 1G13 (for *XNR_3174*), 2C15 (for *XNR_2339*) and 2L15 (for *XNR_4681*) from an *S. albus* ordered genomic library and IMES antibiotic resistance cassettes^39^. The cassette was excised from a carrier plasmid with *Pvu*II and amplified using 3174_F/3174_R, 4681_F/4681_R and 2339_F/2339_R primers Red/ET was performed as previously described^54^. Deletions were confirmed by PCR using the primer pairs 3174C_F1/3174C_R1, 4681C_F/4681C_R and 2339C_F/2339C_R. The recombinant BACs were introduced into the *S. albus* strains. The double-crossover mutants were screened on MS medium supplemented with apramycin or hygromycin and 50 μg/mL of X-Gluc. The resistance marker was removed from the chromosome of the generated mutants by expression of øC31 actinophage integrase^39^, and the resulting strain genotypes were confirmed by PCR with the above-described primers.

### Construction of complementation plasmids

*XNR_3174* was amplified from the genomic DNA of *S. albus* J1074 using the primer pair 3174E_BamHIF/3174E_HindIIIR. The PCR product was digested with *Bam*HI*/Hind*III and cloned into pUWLHFLP (Dr. Fedoryshyn, personal communication), replacing the *flp* gene and resulting in pUWLH3174. *XNR_2339* was amplified with the primer pair 2339E_F/2339E_R. The amplified fragment was digested with *Bam*HI*/Hind*III and ligated into pUWLoriT^55^, resulting in pUWLT2339.

### Transcriptional fusion of XNR_2339 promoter to the *gusA* gene

The promoter region of *XNR_2339* was amplified with the primer pair GUS-XbalF/GUS-KpnIR. The amplified fragment was digested with *Kpn*I/*Xba*I and cloned into a *Kpn*I/*Xba*I-digested pGUS-MF-PVV plasmid. The resulting construct was assigned as pGUS*P*aco. The plasmid pGUS*P*aco was introduced into *S. albus* J1074 and *S. albus* Δ3174. The β-glucuronidase activity was tested as described above^31^.

### Metabolite extraction and analysis

*S. albus* strains were grown in 10 mL of TSB for 1 day, and 1 mL of each culture was used to inoculate the 50 mL of production media. Cultures were grown for 5 days at 30 °C. Metabolites were extracted with ethyl acetate from the supernatant and the acetone:methanol (1:1) mixture from biomass. Extracts were evaporated and dissolved in methanol. 1 µL of each sample was separated using a Dionex Ultimate 3000 UPLC (Thermo Fisher Scientific, USA) and a 10-cm ACQUITY UPLC® BEH C18 column, 1.7 μm (Waters, USA) and a linear gradient of acetonitrile against 0.1% formic acid solution in water from 5% to 95% in 10 or 18 minutes at a flow rate of 0.6 mL/min. Samples were analyzed using an amaZon speed mass spectrometer or, when needed, maXis high-resolution LC-QTOF system (Bruker, USA). Pure ikarugamycin was used as standard in MS/MS experiments (Sigma-Aldrich, USA).

### Isolation and structure elucidation of compounds

*S. albus* ATGSal2P2::Tn14 was grown at 30 °C for 3 days in 6 × 500-mL flasks containing 50 mL of TSB, and pre-culture was used to inoculate 100 × 500-mL flasks containing 50 mL of NL19 media. Cultures were incubated at 30 °C for 5 days. Metabolites were extracted as described above. The extracts from biomass and the supernatant were combined and fractionated by size-exclusion chromatography on an LH 20 Sephadex column (Sigma-Aldrich, USA) using methanol as the solvent. The fractions were collected every 15 minutes., evaporated and dissolved in 0.5 mL of MeOH. Samples were further separated by preparative HPLC (Dionex UltiMate 3000, Thermo Fisher Scientific, USA) using a NUCLEODUR® C18 HTec column (250 × 10 mm, 5 µm) (Macherey-Nagel, Germany) with a linear gradient of solvent B (acetonitrile with 0.1% of formic acid) against solvent A (water with 0.1% of formic acid) at a flow rate of 4.5 mL/min at 45 °C. Compounds were separated using a gradient starting from 30% and increasing to 70% of B over 30 min. UV spectra were recorded with a DAD detector at 280 nm. Individual peaks were collected and analyzed by LC-MS as described above.

NMR spectra were acquired on a Bruker Ascend 700 MHz NMR spectrometer equipped with a 5 mm TXI cryoprobe (Bruker, USA). Deuterated CDCL_3_ was used as a solvent and HSQC, HMBC and ^1^H-^1^H COSY spectra were recorded using standard pulse programs (Table 5S).

### Expression of heterologous secondary metabolism gene clusters

Constructs carrying gene clusters for biosynthesis of pamamycin, griseorhodin, aranciamycin and tunicamycin (Table 1S) were transferred into *S*. albus J1074 and *S. albus* Δ3174 by mean of intergeneric conjugation. Three independent clones of each recombinant strain were grown in 10 mL of TSB for 2 days and 200 mg of biomass of each pre-culture was used to inoculated 50 mL of production media (NL19, SM17 and SG or SGG). Strains were cultivated for 5 days at 28 °C and metabolites were extracted and analyzed as described above. Metabolite accumulation was averaged and normalized by biomass. The production by *S. albus* J1074 was taken as 100% in each particular experiment.

### Secondary metabolism gene clusters transcription analysis

Multiple *S. albus* J1074 RNAseq experimental datasets generated in our laboratory over the last 5 years were used to compare biosynthetic gene cluster expression across multiple media/conditions. First, gene RPKM expression values were ranked from highest to lowest and expressed as percentiles within each sample. The resulting values from all samples were combined into a single summary table for comparison. Conversion to percentiles was used as the most reliable normalization technique for comparing data from different experiments and sequencing providers. This process loses most of the information about gene expression (absolute values and fold-changes within samples) but maintains the relative order of ranked gene expression, which is sufficient for basic qualitative comparisons. To verify that the percentile yield is sufficiently robust for cross-experimental comparison, the β-subunit of the RNA polymerase gene (*XNR_3712*) was included. To accurately depict the meaning of percentile expression, RPKM values within each sample were normalized to 100% and plotted (after log10 transformation of the RPKM % axis) together with percentile values.

## Electronic supplementary material

supplementary information
