# Supplementary material for: Identification of butenolide regulatory system controlling secondary metabolism in Streptomyces albus J1074
Source: Sci Rep. 2017 Aug 29;7:9784. doi: 10.1038/s41598-017-10316-y (PMC5575351; doi:10.1038/s41598-017-10316-y)
Supplement: Supplementary file 1 — supplementary information [file 41598_2017_10316_MOESM1_ESM.pdf]

Supplementary information.

Identification of butenolide regulatory system controlling secondary metabolism in Streptomyces albus J1074

Yousra Ahmed[a], Yuriy Rebets[a,b], Bogdan Tokovenko[a1], Elke Brötz[a2], Andriy Luzhetskyy[a,b]*

[a]Helmholtz-Institute for Pharmaceutical Research Saarland, Actinobacteria Metabolic Engineering Group, Building E8.1, 66123 Saarbrücken, Germany.

[b]Universität des Saarlandes, Pharmazeutische Biotechnologie, Building C2.3, 66123 Saarbrücken, Germany.

Yousra.Ahmed@helmholtz-hzi.de

Yuriy.Rebets@helmholtz-hzi.de

to.bogdan@gmail.com

elidigo@t-online.de

a.luzhetskyy@mx.uni-saarland.de

Corresponding author:

Prof. Dr. Andriy Luzhetskyy

E-mail: a.luzhetskyy@mx.uni-saarland.de

Universität des Saarlandes, Pharmazeutische Biotechnologie, Building C2.3, 66123 Saarbrücken (Germany)

Telephone: +49 681 302-70215

Present addresses:

[1]BASF SE, GBW/H -67056 Ludwigshafen am Rhein, Germany.

[2]Weilburger Coatings GmbH, Ahäuser Weg 12-22, 35781 Weilburg/Lahn, Germany.

Table 1S. Strains and plasmids used in this work.

Strains	**Features**	**Reference or source**
Streptomyces strains		
S. albus J1074	Wild-type strain	[1]
S. albus ATGSal2P2	*S. albus* J1074 harboring pGUSATGSal2P2 construct	This work
S. albus ATGSal2P2::TN1	*S. albus* ATGSal2P2 strain with *Himar1* minitransposon insertion	This work
S. albus ATGSal2P2::TN5	*S. albus* ATGSal2P2 strain with *Himar1* minitransposon insertion	This work
S. albus ATGSal2P2::TN13	*S. albus* ATGSal2P2 strain with *Himar1* minitransposon insertion	This work
S. albus ATGSal2P2::TN14	*S. albus* ATGSal2P2 strain with *Himar1* minitransposon insertion	This work
S. albus Δ3174	*S. albus* J1074 strain with deletion of *XNR_3174* gene	This work
S. albus Δ3174 pUWLH3174	*S. albus* Δ3174 strain harboring pUWLH3174 construct	This work
S. albus Tn14Δ2339	*S. albus* ATGSal2P2Tn14 strain with deletion of *XNR_2339* gene	This work
S. albus Tn14Δ2339 pUWLT2339	*S. albus* ATGSal2P2Tn14 Δ2339 strain with pUWLT2339 construct	This work
S. albus Δ3174 Δ2339	*S. albus* Δ3174 strain with deletion of *XNR_2339* gene	This work
S. albus J1074 pGUS*P*aco	*S. albus* J1074 strain with pGUS*P*aco construct	This work
S. albus Δ3174 pGUS*P*aco	*S. albus* Δ3174 with pGUS*P*aco construct gene	This work
S. albus Δ4681	*S. albus* J1074 strain with deletion of *XNR_4681* gene	This work
E. coli strains		
DH10β	General cloning host	[2]
ET12567 pUB307	Donor strain for intergeneric conjugation	[3]
TransforMaxTMEC100DTM pir-116	*E.coli* with *pir* gene for replication of plasmids containing the *R6Kγ* origin of replication	Epicenter
**Plasmids**		
pHTM	*Himar1* minitransposon delivery plasmid.	Bilyk et al. (2013)
pUWL-Dre	Plasmid containing synthetic *dre(a)* gene under the *tipA* promoter, pSG5rep and *oriT*	Fedoryshyn (2008b)
patt-saac-oriT	Resistance cassette plasmid containing a synthetic fragment with *aac(3)IV*, *oriT*, *B-CC*, *P-GG* and *loxP* sites	Myronovskyi et al. (2014)
patt-shyg-oriT	Resistance cassette plasmid containing a synthetic fragment with *hyg*, *oriT*, *B-CC*, *P-GG* and *loxP* sites	Myronovskyi et al. (2014)
pUWLHFLP	Replicative vector with pIJ101 replicon, *oriT*, *tsr*, *bla* and *hyg* resistance genes with the *flp(a)* gene under an *ermE* promoter	Dr. M. Fedoryshyn, personal communication
pUWLH3174	pUWLHFLP derivative with *XNR_3174* gene cloned under control of *ermE* promoter and replacing *flp* gene	This work
pUWLoriT	Replicative vector with pIJ101 replicon, *oriT*, *tsr*, *bla* resistance genes.	[4]
pUWLT2339	pUWLoriT derivative with *XNR_2339* gene cloned under control of *ermE* promoter	This work

pGUS-MF-PVV	Plasmid containing a synthetic *gusA* gene flanked with transcriptional terminators, *aac(3)IV, aadA, oriT, intøC31*.	Dr. N. Manderscheid, personal communication
pGUSATGSal2P2	pGUS-MF-PVV with promoter of *XNR_0204* gene fused with *gusA* reporter	This work
pGUS*P*aco	pGUS-MF-PVV with promoter of *XNR_2339* gene fused with *gusA* reporter	This work
**BACs**		
pSMART	Cm^R; BAC vector	Lucigen
pSMARTgus	Derivative of pSMART with the *gusA* gene	Dr. M. Myronovskyi, personal communication
1G13	pSMARTgus derivative containing a fragment of the *S. albus* chromosome	[5]
2C15	pSMARTgus derivative containing a fragment of the *S. albus* chromosome	[5]
2L15	pSMARTgus derivative containing a fragment of the *S. albus* chromosome	[5]
1G13am	Derivative of 1G13 with *XNR_3174* gene substituted with a cassette from patt-saac-oriT	This work
2C15hyg	Derivative of 2C15 with *XNR_2339* gene substituted with a cassette from patt-shyg-oriT	This work
2L15am	Derivative of 2L15 with *XNR_4681* gene substituted with a cassette from patt-saac-oriT	This work
p41-2C-06	pOJ436 derivative, containing aranciamycin biosynthetic gene cluster	[6]
PMP31	pOJ436 derivative, containing the griseorhodin biosynthetic cluster	[7]
pIJ12003a	12.9 Kbp *tun*-gene cluster cloned into the pRT802	[8]
pOJR2	pOJ436 with cloned *pam*-gene cluster	[9]

Table 2S. Primers used in this work.

**Primers**	**Sequence**	**Purpose**
Sal2P2F	ATTGGTACCGTTGATCGCGTCAGCCAAGT	*XNR_0204* promoter cloning
Sal2P2R	ATTGATATCTCCACGACCCCACGACGTT	
3174-F	TCCGGCCGGATTCCGCCGGGCCCCGGGCTCAGTCCGTCTATTCCG GGGATCCGTCGACC	Deletion of *XNR_3174*
3174-R	GTTCCTGTAATGCATGACCCTGAGGGGGGTTCTTATGGGTGTAGG CTGGAGCTGCTTC	
2339-F	CATGACTCCCTGGCGACCGTTACTTTACAAAGTGTGATTACTACG CCCCCAACTGAGAG	Deletion of *XNR_2339*
2339-R	CCGTCCCGCGTCCCGCCGTCGTGCGCCTCAGCCCGTCATTCGACC CGGTACCGGAGTA	
3174C_ F1	AGCGGATACAGGCATCATGT	Verification of *XNR_3174* deletion
3174C_R1	GGAAGCAGTACGTACTGCTT	
2339C_F	ACTGGCTTGAACTGGTCACT	Verification of *XNR_2339* deletion
2339C_R	GGAGGACGAGAGACGCGA	
3174E_HindIIIR	AAAAGCTTGAGGGGGGTTCTTATGGGG	Expression of *XNR_3174*
3174E_BamHIF	AAGGATCCCCCGGGCTCAGTCCGTCT	
2339E-F	TTGGATCCCCGTTACTTTACAAAGTGTG	Expression of *XNR_2339*
2339E-R	TTAAGCTTTGCGCCTCAGCCCGTCAT	
GUS-XbaI F	AATCTAGATTCCGCTTTTCGCCCTGG	*XNR_2339* promoter cloning
GUS-KpnI R	AAGGTACCTGTTCTTCGCCGCGTGTCG	
p3-pALG-ch	GTGAGCCGCCGCGTGCCGTCG	*Himar1* transposon sequencing primer
4681-F	TCGTCACGGAAACGTTGAACGAAGGTGGACAGCACGTTGCTACG CCCCCAACTGAGAG	Deletion of *XNR_4681*
4681-R	GCGATGTCACCGGAGGGAGCCCGGTCGCGTCGTCACTCC TCGACCCGGTACCGGAGTA	
4681C-F	TCGAATGGCCAGCAACCG	Verification of *XNR_4681* deletion
4681C-R	GTCTGCCGACTCCGCTCT	

Table 3S. Expression level of *S. albus* J1074 secondary metabolites gene clusters shown in rpkm and in corresponding percentile rank in different mediums.

BGC number	BGC type	SGG		TSB		NL19		Solid MS		GYM	
		rpkm	Percentile rank	rpkm	Percentile rank	rpkm	Percentile rank	rpkm	Percentile rank	rpkm	Percentile rank
Cluster_1	unknown	10.597	46.526	3.058	21.177	2.126	31.286	1.831	13.708	35.954	57.039
Cluster_2	polycyclic tetramate macrolactam	23.210	63.010	3.625	23.616	20.800	70.613	6.560	32.598	23.071	46.122
Cluster_3	hopene	20.588	60.689	16.047	49.571	8.234	56.551	7.130	33.961	18.388	41.009
Cluster_4	unknown	6.559	36.736	4.809	28.074	3.214	38.923	3.817	23.481	18.531	41.194
Cluster_5	paulomycin	19.600	59.764	22.661	55.929	47.122	80.235	230.764	88.830	32.334	54.348
Cluster_6	unknown	35.982	71.169	22.365	55.609	3.487	40.571	15.207	48.376	56.633	67.014
Cluster_7	glycosylated peptide	9.297	43.767	7.685	36.114	18.849	69.150	34.461	63.498	26.920	49.924
Cluster_8	unknown	40.749	73.120	71.461	76.804	35.077	77.039	71.827	75.828	78.350	73.490
Cluster_9	geosmin	28.826	67.283	39.081	66.223	53.161	81.530	23.295	56.585	36.071	57.123
Cluster_10	albaflavenone	43.595	74.230	78.150	78.015	42.417	79.041	74.640	76.484	131.328	81.984
Cluster_11	class I lantibiotic	108.485	85.601	6.465	33.372	3.392	40	68.239	74.886	2218.697	98.738
Cluster_12	AmfS	31.249	68.595	40.349	66.997	22.141	71.589	121.733	82.539	58.194	67.687
Cluster_13	unknown	18.411	58.317	45.438	69.402	45.012	79.814	29.422	60.723	37.707	58.065
Cluster_14	lipopeptide	4.694	29.083	20.291	53.658	6.787	52.834	40.523	66.156	24.600	47.603
Cluster_15	unknown	7.572	39.562	119.160	83.465	24.220	72.649	350.572	91.740	89.394	76.013
Cluster_16	desferrioxamine	20.763	60.841	16.613	50.142	25.567	73.254	14.813	47.737	44.890	62.035
Cluster_17	ectoine or 5-hydroxyectoine	253.957	91.673	70.066	76.467	225.034	92.178	101.391	80.437	309.959	91.051
Cluster_18	indigoidine	16.376	55.912	22.324	55.542	19.269	69.503	41.066	66.425	40.575	59.663
Cluster_19	linocin M18 family	3.239	22.388	1.307	12.682	0.613	13.978	2.031	14.886	13.297	33.439
Cluster_20	isorenieratene	12.790	50.647	5.825	31.337	32.296	76.164	8.580	37.611	17.552	39.798
Cluster_21	THN, flaviolin	16.721	56.383	3.770	24.205	5.386	48.107	4.434	25.735	30.809	53.372
Cluster_22	candicidins	14.99	54.028	40.574	67.182	2.204	31.841	18.302	51.959	12.465	31.724
Cluster_23	antimycins	18.352	58.200	64.728	75.323	2.072	30.782	42.134	66.879	8.968	24.642
Cluster_24	unknown	4.002	26.274	7.391	35.306	4.300	44.592	3.880	23.616	36.695	57.460

Cluster_25	class II lantibiotic	1.446	11.404	2.187	17.493	1.188	21.883	1.831	13.708	17.851	40.218
Cluster_26	unknown	6.541	36.585	6.386	33.120	9.171	58.250	3.966	23.851	97.951	77.577
XNR_2339	Acyl-CoA oxidase	4.890	29.840	41.735	67.838	7.540	54.936	29.637	60.807	43.522	61.429
XNR_2340	Cytochrome P450	1.376	10.714	79.044	78.149	6.334	51.370	21.828	55.340	10.148	27.333
XNR_2800	Type IV secretory pathway	14.622	53.439	15.078	48.544	25.947	73.557	3.162	20.521	39.950	59.461
XNR_3174	LuxR-family transcriptional regulator	7.411	39.243	9.155	39.310	83.671	85.668	41.502	66.526	51.625	65.214
XNR_4681	Gamma butyrolactone receptor protein	14.817	53.725	30.920	61.833	51.857	81.211	71.149	75.727	21.527	44.524
XNR_3521	excisionase/Xis, DNA-binding protein	8641.688	–	1792.553	–	9451.419	–	5309.298	–	15730.04	–
XNR_3712	DNA-directed RNA polymerase beta subunit	506.409	94.768	737.395	95.559	1504.089	98.233	448.551	93.355	796.080	96.164
XNR_3720	30S ribosomal protein S12	2683.501	98.519	3634.723	98.671	9949.691	99.798	2336.997	98.502	2395.937	98.940

Table 4S. Localization of transposon insertion in the chromosome of selected *S. albus* ATGSal2p2 mutants

**Strain name.**	**Gene ID.**	**Position of insertion.**	**Putative gene product.**
ATGSal2p2::Tn1	*XNR_2800*	3,180,172	Putative type IV secretory pathway
	XNR_3855	4,381,847	Putative glutaminase
ATGSal2p2::Tn5	*XNR_4186*	4,745,403	Putative hypothetical protein upstream
ATGSal2p2::Tn13	*XNR_3521*	4,038, 469	Putative MerR family transcriptional regulator
ATGSal2p2::Tn14	*XNR_3174*	3,624,446	Putative LuxR family transcriptional regulator

Table 5S. NMR spectroscopic data for butenolide 4 in $CDCl_3$ (700 MHz, 25 °C)

**Pos.**	δ_C	δ_H**, mult, (*J* in Hz)**	**COSY**	**HMBC**
1	173.4	-	-	2-H, 3-H
2	121.9	6.10, dd (5.7, 1.7)	4-H, 3-H	^1J, 4-H, 3-H
3	156.5	7.43, ddd (5.5, 5.7, 1.4)	2-H, 4-H	^1J, 5-H_2, 2-H, 4-H
4	83.6	5.02, ddd (5.5, 1.7, 7.0)	2-H, 3-H, 5-H_2	5-H_2, 2-H, 3-H
5	33.2	1.76, m<br>1.64, m	5-H_b, 4-H, 6-H_2<br>5-H_a, 4-H, 6-H_2	4-H, 6-H_2, 7-H_2
6	25.0	1.44, m	(5-H_a)	^1J, 4-H, 5-H_2, 7-H_2,
7	29.5	1.33, m	8-H, 9-H_a	5-H_b, 9-H_a
8	27.1	1.25, m	7-H_2, 9-H_b	7-H_2, 9-H_a, 10-H
9	32.8	1.64, m<br>1.31, m	9-H_b, 8-H, 10-H<br>9-H_a, 10-H	8-H_2, 10-H, 13-H_3
10	47.3	2.49, m	9-H_2, 13-H_3	9-H_2, 12-H_3, 13-H_3
11	212.6	-	-	9-H_2, 10-H, 12-H_3, 13-H_3
12	28.2	2.13, s	-	^1J
13	16.5	1.08, d (7.2)	10-H	^1J, 9-H_2, 10-H

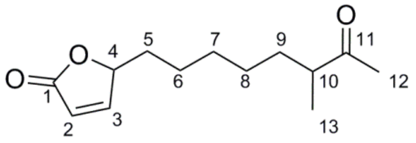

Compound Y3 (0.4 mg) was obtained as oily substance and showed molecular ion *m/z* = 225.1483 $[M+H]^+$ corresponding to a molecular formula of $C_{13}H_{21}O_3$ for the protonated ion, UV (AcN) λmax. 208 nm. NMR data and literature survey determined the compound as 5-(6-Methyl-7-oxooctyl)-2(*5H*)-furanone. The chemical shift δ_C = 173,4 ppm is pointing on carbonyl group at C-1. C-1 C-1 is monitoring C-1/2-H and C1/3-H HMBC cross-peaks showing the connection of C-1 to the methines which couple to each other in ^{1}H spectrum ^3J = 5,7 Hz and in COSY spectrum (2-H/3-H; 3-H/2-H). Further 2-H shows COSY cross-peak and ^4J = 1,7 Hz to 4-H. 3-H shows COSY cross-peaks 3-H/4-H and ^3J = 5,5 Hz to 4-H. Methine C-4 (δ_C = 83,6 ppm)

supports the furanone structure (HMBC signals 4-H/3-H and 4-H/2-H). The side-chain at C-4 is defined by COSY (4-H/5-H_2) and HMBC (C-4/5-H_2) signals. Methylenes 5-H_2–9-H_2 in the chain shows multiplet signals due to coupling to their neighbors. COSY and HMBC signals lead through the chain sequence. The first methylene group 5-H_2 (δ_H = 1,76 ppm and 1,64 ppm) shows COSY cross-peaks 5-H_2/6-H_2, 5-H_2/4-H and HMBC-signals C-5/6-H_2 and C-5/7-H_2. 7-H_2 monitors HMBC signal C-7/5-H_2 supporting the connection to the left chain sequence and HMBC signal C-7/9-H_2 in combination with COSY cross-peaks to 8-H_2 and 9-H_2 supporting the connection to the right chain sequence. Methylene group 9-H_2 (δ_H = 1,64 ppm and 1,31 ppm) shows COSY coupling 9-H_2/10-H and the HMBC signal C-9/13-H_3. 13-H_3 methyl group (δ_C = 16,5 ppm) shows a doublet with ^3J = 7,2 Hz and a COSY cross-peak 13-H3/10-H determining the 10-H as a neighbor. The HMBC signal C-13/9-H_2 clearly assigns the connection to the chain. The positon of the carbonyl (δ_C = 212,6 ppm) is determined based on several HMBC signals C-11/9-H_2, C-11/10-H and C-11/13-H_3. Finally the single methyl group C-12 is showing no coupling but the position is supported by HMBC signals C-10/12-H_3 and C-11/12-H_3.

Table 6S. BLAST search for homologues of Aco and Cyp17 proteins from *S. avermitilis*, XNR_2339, XNR_2340 and XNR_3174 from *S. albus* J1074, A-factor synthase AfsA from *S. griseus*, frontalamide, antimycin and candicidin biosynthesis gene clusters within the actinobacteria genomes available from public databases. The core genes from each gene cluster were used for homologues search: polycyclic tetramate macrolactams (PTL) – XNR_0204 (PKS-NRPS); candicidins – XNR_5854 (PKS I); antimycins – XNR_5891 (AntC NRPS).

**Strain**	**Aco**	**Cyp17**	**XNR_2339**	**XNR_2340**	**XNR_3174**	**AfsA**	**PTM**	**Antimycins**	**Candicidins**
***Streptomyces albus* sub-group**									
Streptomyces albus J1074	51/60	54/67	100	100	100	-	100	100	99/99
Streptomyces sp. NRRL B-3253	49/60	46/60	99/100	99/99	99/100	-	99/99	99/99	55/66
Streptomyces albidoflavus NRRL B-1271	49/60	46/60	99/99	99/99	99/100	-	99/99	100/100	57/66
Streptomyces sp. LaPpAH-202	49/60	46/60	99/99	99/99	99/100	-	99/99	99/99	99/99
Streptomyces sp. S4	49/60	46/60	99/99	99/100	99/100	40/60	99/99	99/99	99/98
Streptomyces sp. SM8	50/60	45/57	98/98	100	100/100	38/48	99/99	98/98	99/99
Streptomyces sp. NRRL F-6628	49/60	46/60	99/100	100	99/100	-	99/99	-	-
Streptomyces sp. CNY228	49/60	46/60	97/97	100	99/100	39/50	99/99	99/99	99/99
Streptomyces wadayamensis	49/60	46/60	100	100	99/100	42/58	99/99	-	-
Streptomyces sp. CNQ431	49/60	46/60	95/97	99/99	99/99	40/52	97/97	95/96	-
Streptomyces sp. PVA 94-07	49/60	46/60	95/97	99/99	99/99	40/52	96/97	99/99	96/97
Streptomyces sp. GBA 94-10	49/60	46/60	95/97	99/99	99/99	40/52	97/97	99/99	96/97
Streptomyces sp. KE1	50/60	46/60	96/97	98/99	99/99	-	97/98	97/97	97/98
Streptomyces sp. M10	49/60	46/57	95/96	98/98	99/99	43/51	97/98	96/97	-
Streptomyces sampsonii	46/60	49/60	98/98	100/100	99/100	-	99/99	99/99	99/99
Streptomyces sp. FR-008	49/60	46/60	98/98	100/100	99/100	-	99/99	99/99	98/99
Streptomyces sp. ScaeMP-6W	49/59	46/60	98/98	100/100	100	-	99/99	99/99	98/99
Streptomyces sp. IgraMP-1	49/60	46/60	99/99	100/100	100	-	99/99	99/99	99/99
Streptomyces sp. BvitLS-983	49/60	46/60	99/99	100/100	100	-	99/99	99/99	98/98
***Streptomyces avermitilis* sub-group**									
Streptomyces avermitilis MA-4680	100	100	59/67	37/55	58/73	34/47	n.a.	n.a.	n.a.
Streptomyces aurantiacus	77/82	74/81	52/62	48/61	59/74	no	n.a.	n.a.	n.a.
Streptomyces cyaneogriseus	74/82	70/78	48/59	37/51	56/72	39/48	n.a.	n.a.	n.a.
Streptomyces sp. NRRL F-5727	72/81	61/74	55/64	37/52	68/84	45/54	n.a.	n.a.	n.a.
Streptomyces showdoensis	71/80	65/76	51/60	35/53	64/77	38/49	n.a.	n.a.	n.a.
Streptomyces sp. WM6378	66/75	72/82	52/63	35/52	67/82	38/51	n.a.	n.a.	n.a.
Streptomyces griseoaurantiacus	66/72	72/80	52/62	70/79	60/76	no	n.a.	n.a.	n.a.
Streptomyces sp. XY332	66/73	67/78	54/66	37/51	62/77	no	n.a.	n.a.	n.a.
Streptomyces aureofaciens	65/75	66/76	52/62	51/65	56/70	43/54	n.a.	n.a.	n.a.
Streptomyces glaucescens	64/71	65/77	53/63	37/54	59/76	37/48	n.a.	n.a.	n.a.
Streptomyces neyagawaensis	63/71	64/77	53/65	49/64	56/72	31/40	n.a.	n.a.	n.a.
Streptomyces viridosporus	63/75	61/74	49/62	77/85	59/72	40/51	n.a.	n.a.	n.a.

Streptomyces leeuwenhoekii	63/74	70/79	49/60	38/52	56/72	39/49	n.a.	n.a.	n.a.
Streptomyces ghanaensis	63/75	61/74	50/65	77/85	59/73	40/51	n.a.	n.a.	n.a.
Streptomyces sp. NRRL B-3229	62/71	63/75	52/61	48/61	56/72	41/50	n.a.	n.a.	n.a.
Streptomyces torulosus	62/73	59/70	52/65	50/65	55/72	38/50	n.a.	n.a.	n.a.
Streptomyces collinus	61/67	61/75	53/63	50/64	61/75	no	n.a.	n.a.	n.a.
Streptomyces kanamyceticus	61/69	68/78	51/61	43/57	59/74	35/48	n.a.	n.a.	n.a.
Streptomyces sp. LaPpAH-108	60/68	63/78	54/63	45/59	54/70	39/50	n.a.	n.a.	n.a.
Streptomyces azureus	60/68	54/65	51/62	42/54	56/71	43/54	n.a.	n.a.	n.a.
Streptomyces ossamyceticus	60/72	59/71	52/64	53/70	56/72	no	n.a.	n.a.	n.a.
Streptomyces fradiae	58/67	64/75	55/65	56/68	-	42/53	n.a.	n.a.	n.a.
Streptomyces sp. NRRL S-1868	54/63	59/69	48/58	47/61	-	-	n.a.	n.a.	n.a.
Streptomyces sp. NRRL F-5053	54/63	59/69	48/58	47/61	-	no	n.a.	n.a.	n.a.
Streptomyces sp. Root264	54/63	63/74	49/61	51/66	56/73	31/40	n.a.	n.a.	n.a.
Streptomyces bambergiensis	53/63	48/59	68/77	75/83	77/85	43/51	n.a.	n.a.	n.a.
Streptomyces prasinus	53/63	44/56	68/77	75/83	54/69	42/50	n.a.	n.a.	n.a.
Streptomyces sviceus ATCC 29083	52/62	39/50	68/76	67/78	56/72	42/53	n.a.	n.a.	n.a.
Streptomyces canus	51/61	41/52	67/76	68/78	56/72	39/49	n.a.	n.a.	n.a.
Streptomyces mirabilis	60/73	62/73	67/76	69/79	59/72	42/50	n.a.	n.a.	n.a.
Streptomyces prasinopilosus	61/71	44/56	67/78	75/82	54/69	33/45	n.a.	n.a.	n.a.
Streptomyces hirsutus	53/63	47/59	66/76	75/83	60/75	-	n.a.	n.a.	n.a.
Streptomyces cyanoalbus	61/72	45/57	66/77	75/83	52/70	-	n.a.	n.a.	n.a.
Streptomyces sp. Root369	52/62	38/50	66/76	69/79	58/72	40/50	n.a.	n.a.	n.a.
Streptomyces sp. NBRC 110035	61/71	43/54	65/75	75/83	60/75	35/45	n.a.	n.a.	n.a.
Streptomyces scopuliridis	54/64	46/58	64/73	55/69	63/77	42/55	n.a.	n.a.	n.a.
Streptomyces hygroscopicus	52/62	54/66	63/73	77/83	59/73	34/46	n.a.	n.a.	n.a.
Streptomyces sp. W007	51/60	46/59	63/74	63/74	59/74	86/89	n.a.	n.a.	n.a.
Streptomyces cyaneofuscatus	49/60	45/58	62/72	64/75	57/74	72/79	n.a.	n.a.	n.a.
Streptomyces sp. CcalMP-8W	61/70	46/58	62/71	65/76	58/74	70/78	n.a.	n.a.	n.a.
Streptomyces sp. SolWspMP-sol2th	61/70	46/58	62/72	65/76	57/74	70/78	n.a.	n.a.	n.a.
Streptomyces sp. ScaeMP-e10	50/60	46/58	62/72	63/75	60/75	72/79	n.a.	n.a.	n.a.
Streptomyces alboviridis	50/61	45/56	62/73	64/76	58/73	73/81	n.a.	n.a.	n.a.
Streptomyces sp. NRRL S-31	52/64	53/66	62/73	77/85	57/72	40//50	n.a.	n.a.	n.a.
Streptomyces mediolani	48/59	54/68	62/72	63/75	61/77	79/88	n.a.	n.a.	n.a.
Streptomyces fulvissimus DSM 40593	50/61	45/56	62/73	62/74	58/73	73/80	n.a.	n.a.	n.a.
Streptomyces europaeiscabiei	62/72	71/81	62/73	63/74	58/72	85/89	n.a.	n.a.	n.a.
Streptomyces sp. NRRL S-623	50/60	46/58	61/72	64/77	55/72	73/80	n.a.	n.a.	n.a.
Streptomyces anulatus	52/60	45/58	61/72	64/75	59/74	85/88	n.a.	n.a.	n.a.
Streptomyces sp. CNS654	51/60	45/56	61/73	64/75	59/75	83/88	n.a.	n.a.	n.a.
Streptomyces sp. JS01	51/59	45/56	61/72	62/73	59/75	81/88	n.a.	n.a.	n.a.
Streptomyces sp. TAA040	53/63	44/56	61/69	65/75	no	no	n.a.	n.a.	n.a.
Streptomyces sp. NRRL S-1022	54/66	48/62	61/73	78/86	58/73	33/47	n.a.	n.a.	n.a.
Streptomyces luridiscabiei	49/60	46/58	61/72	64/77	55/73	72/80	n.a.	n.a.	n.a.
Streptomyces sp. NRRL S-350	56/69	48/60	59/73	62/71	37/54	40/51	n.a.	n.a.	n.a.

- not found; n.a. – not analysed.

% of amino acid identity/similarity

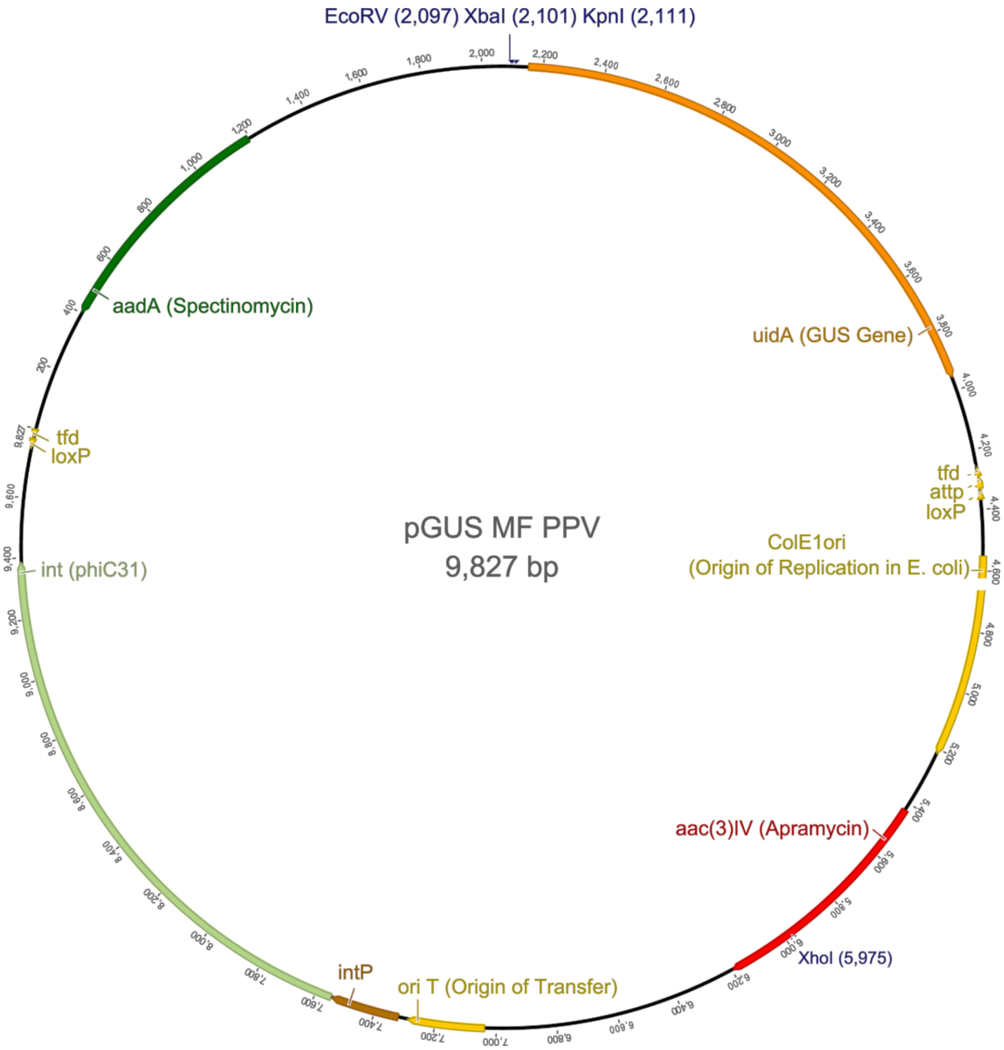

Figure 1S. Map of the plasmid pGUS MF PPV used in this work (Dr. Niko Manderscheid, personal communication). The plasmid carries the reporter gene *gusA* coding for β-glucuronidase with the ATG start codon. Components of the vector are shown with arrows: *uidA* – synthetic *gusA* gene with codon usage adapted for actinobacteria [10]; *aac(3)IV* – apramycin resistance gene; *aadA* – spectinomycin resistance gene; *oriT* – origin of transfer; *attP* – phage øC31 attachment site; *int* – phage øC31 integrase gene; *loxP* – Cre recombinase recognition sites. The reporter construct is flaked with the t_{fd} transcriptional terminators.

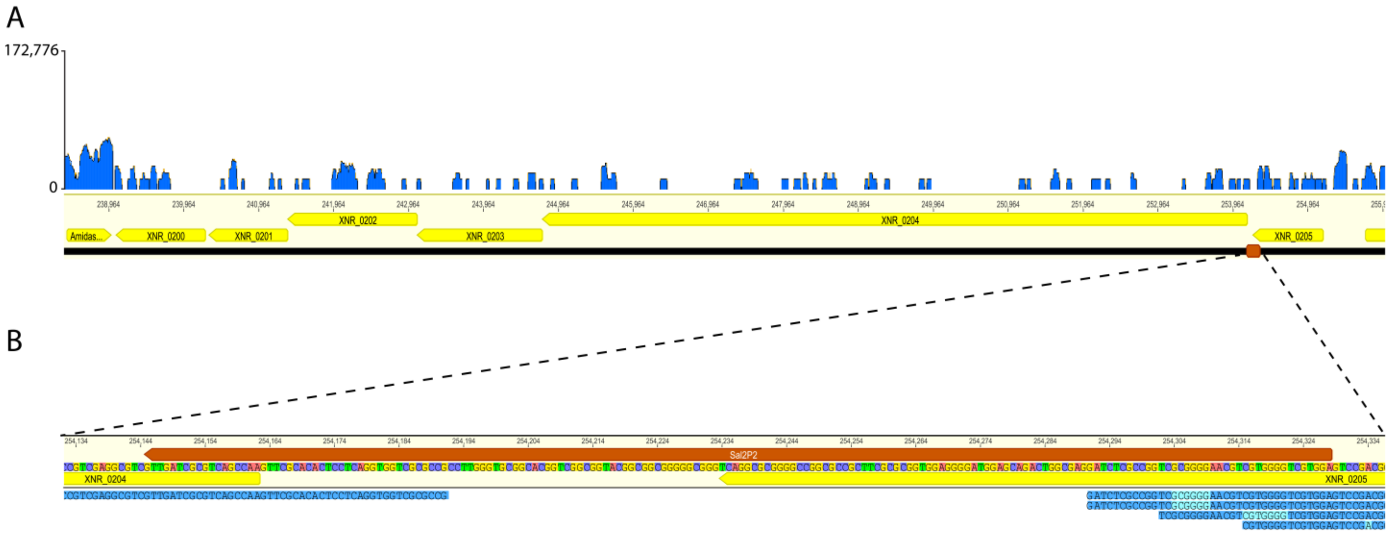

Figure 2S. Transcriptional profile of the *S. albus* J1074 secondary metabolites biosynthesis gene cluster 2 based on RNAseq data. The gene cluster is poorly transcribed in all tested conditions resulting in a very low number of reads mapped. The data used in this experiment was obtained from *S. albus* J1074 culture grown in NL19 medium. **A.** Overall organization of BGC 2. Yellow arrows indicate the size and location of individual genes. The coordinates of the region within the *S. albus* genome are shown above the arrows. The blue plot on top shows the coverage (the number of non-end gap characters at each position). The scale bar indicates the mean coverage level (log scale). The highest coverage is shown. **B.** Zoomed-in representation of *XNR_0204* gene promoter region. Fragment, cloned into the promoter-probing vector is indicated with brown arrow. Sequences highlighted in blue show the RNAseq reads mapped to the corresponding region of the *S. albus* genome. The data analysis, reads mapping and visualization was performed using Geneious software, version 8.1.7 (Biomatters Ltd, New Zealand).

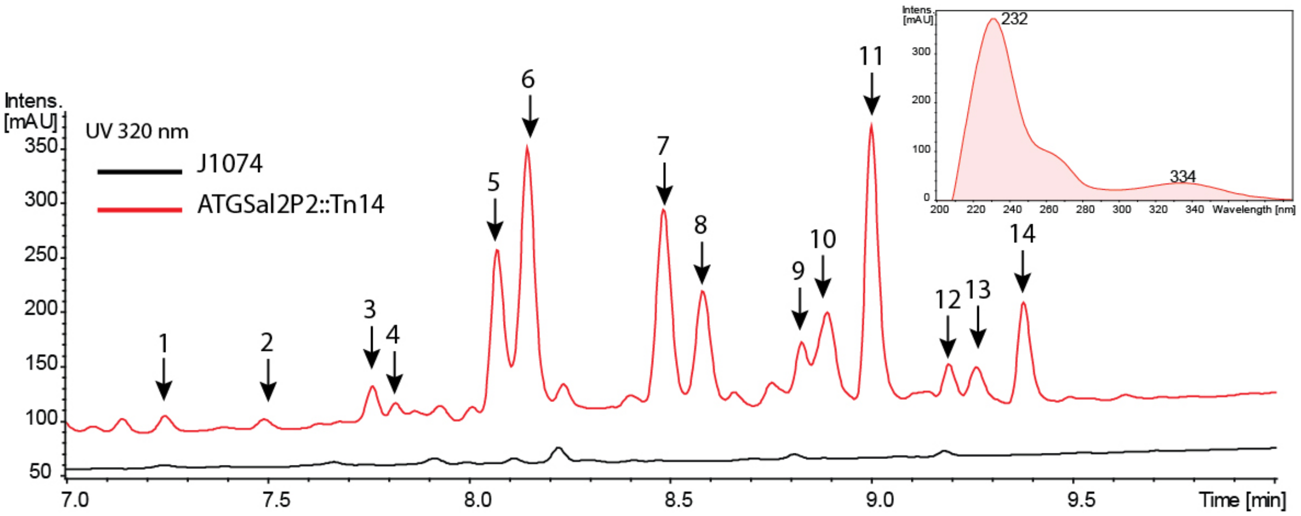

Peak	Antimycin	RT, min	UV, nm	Detected m/z $[M+H]^+$	Calculated m/z $[M+H]^+$
1	A5x	7.3	-	493.2182	493.2180
2	A6x	7.5	-	479.2172	479.2024
3	A3x	7.7	228/330	521.2577	521.2493
4	A10/A14/A15	7.8	228/330	563.3068	563.2963
5	A3x	8.1	230/328	521.2545	521.2493
6	A2 or A11	8.2	230/316	535.2667	535.2650
7	A1/A12/A13/A19	8.5	230/316	549.2951	549.2806
8	A4x/A20	8.6	230/330	507.2567	507.2337
9	A10/A14/A15	8.8	228/330	563.3042	563.2963
10	A1/A12/A13/A19	8.9	230/330	549.2854	549.2806
11	A4x/A20	9.0	232/324	507.2422	507.2337
12	A10/A14/A15	9.2	230/328	563.3353	563.2963
13	A10/A14/A15	9.3	230/328	563.3356	563.2963
14	A3x	9.4	230/330	521.2658	521.2493

Figure 3S. LC-MS chromatogram (at 330 nm) of secondary metabolites extracts of *S. albus* J1074 and *S. albus* ATGSal2p2::Tn14 strains grown in NL19 medium. Samples were separated with the 10 min gradient protocol (see Materials and Methods). The arrows indicate different species of antimycins A, listed in the table below.

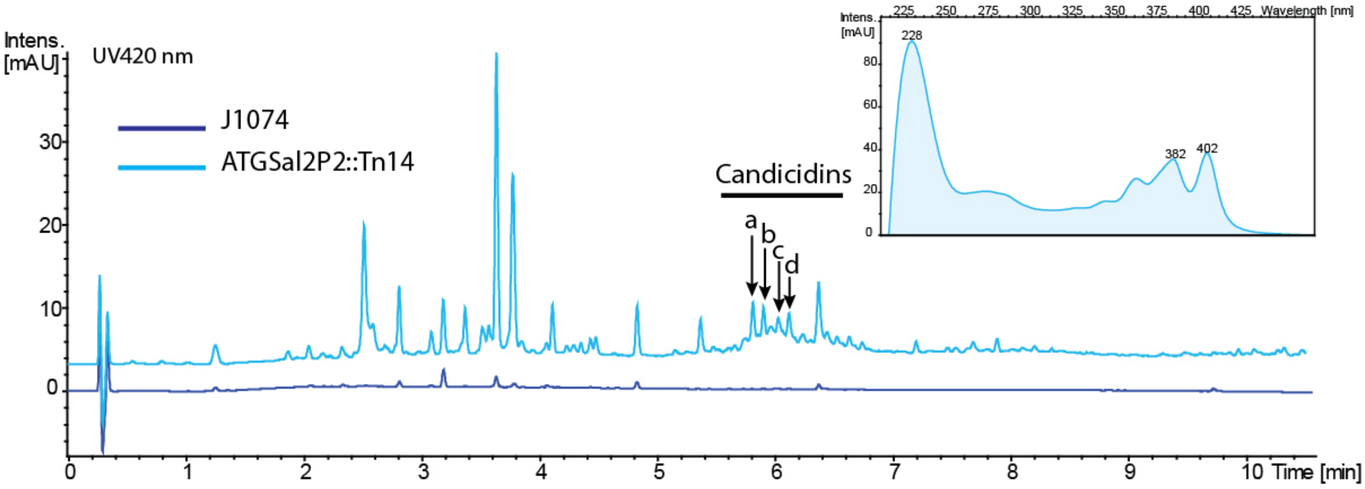

Figure 4S. LC-MS chromatogram (at 420 nm) of secondary metabolites extracts of *S. albus* J1074 and *S. albus* ATGSal2p2::Tn14 strains grown in NL19 medium. Samples were separated with the 10 min gradient protocol (see Materials and Methods). Compounds identified as candicidins are indicated by arrows and marked as: **a** – candicidin I (detected m/z 1111.59 $[M+H]^+$, calculated m/z 1111.5952 $[M+H]^+$), **b** and **c** – candicidins II and III (detected m/z 1109.58 $[M+H]^+$, calculated m/z 1109.5792 $[M+H]^+$), and **d** – candicidin IV (detected m/z 1093.58 $[M+H]^+$, calculated m/z 1093.5842 $[M+H]^+$). Detected m/z values corresponds to the described one for candicidins.

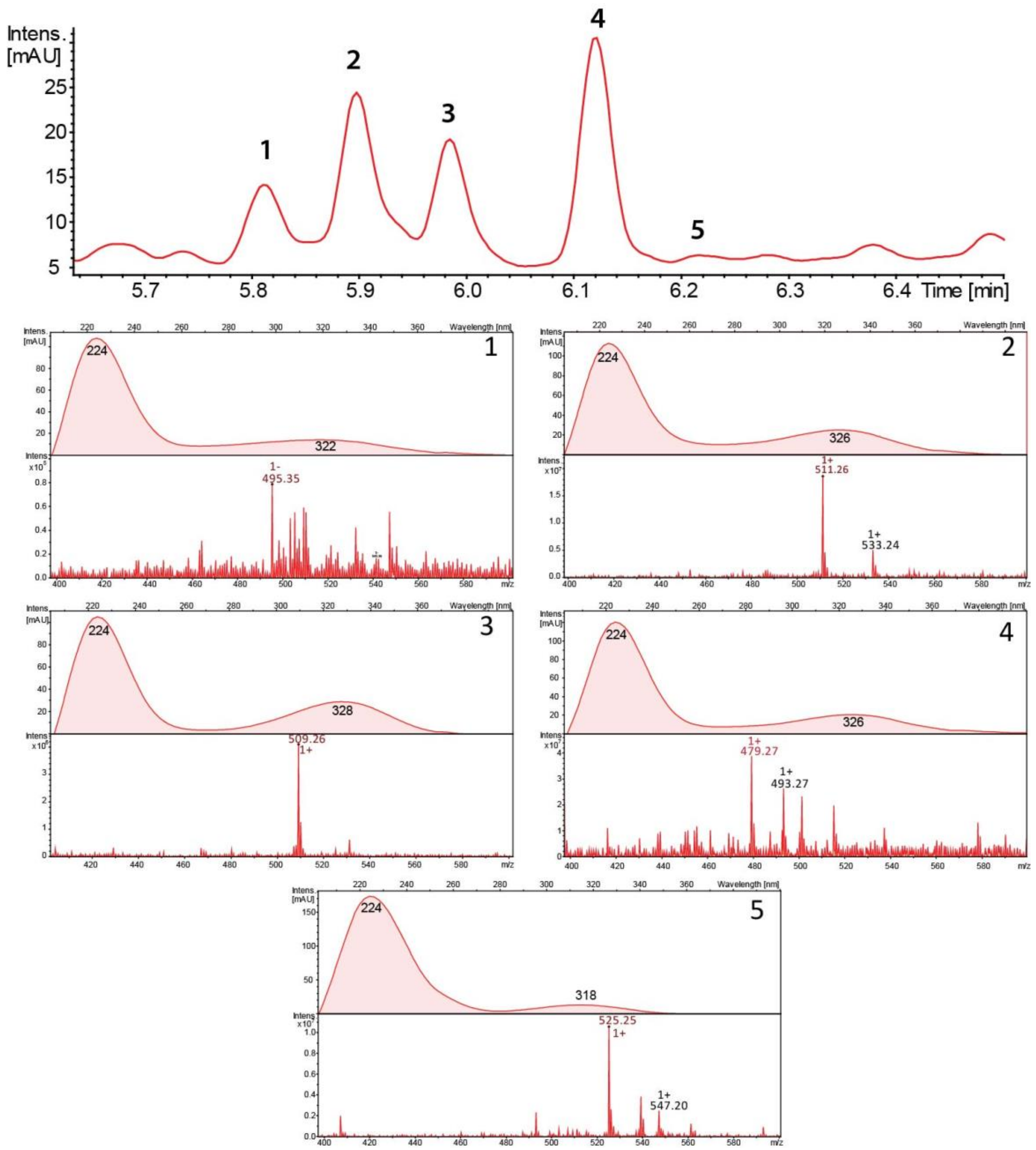

Figure 5S. LC-MS based identification of polycyclic tetramate mactolactams produced by the *S. albus* ATGSal2P2::Tn14 strain. Initial extract from 5L of *S. albus* ATGSal2P2::Tn14 culture (concentrated x 250 folds to final volume of 20 ml in methanol) grown in NL19 medium was fractionated by size exclusion chromatography and collected fractions were analyzed by LC-MS with the 10 minutes gradient protocol (see Materials and Methods). Fraction 11 (shown; chromatogram at 320 nm) contains several compounds with spectral characteristics typical for polycyclic tetramate mactolactams. The m/z values are shown for each peak.

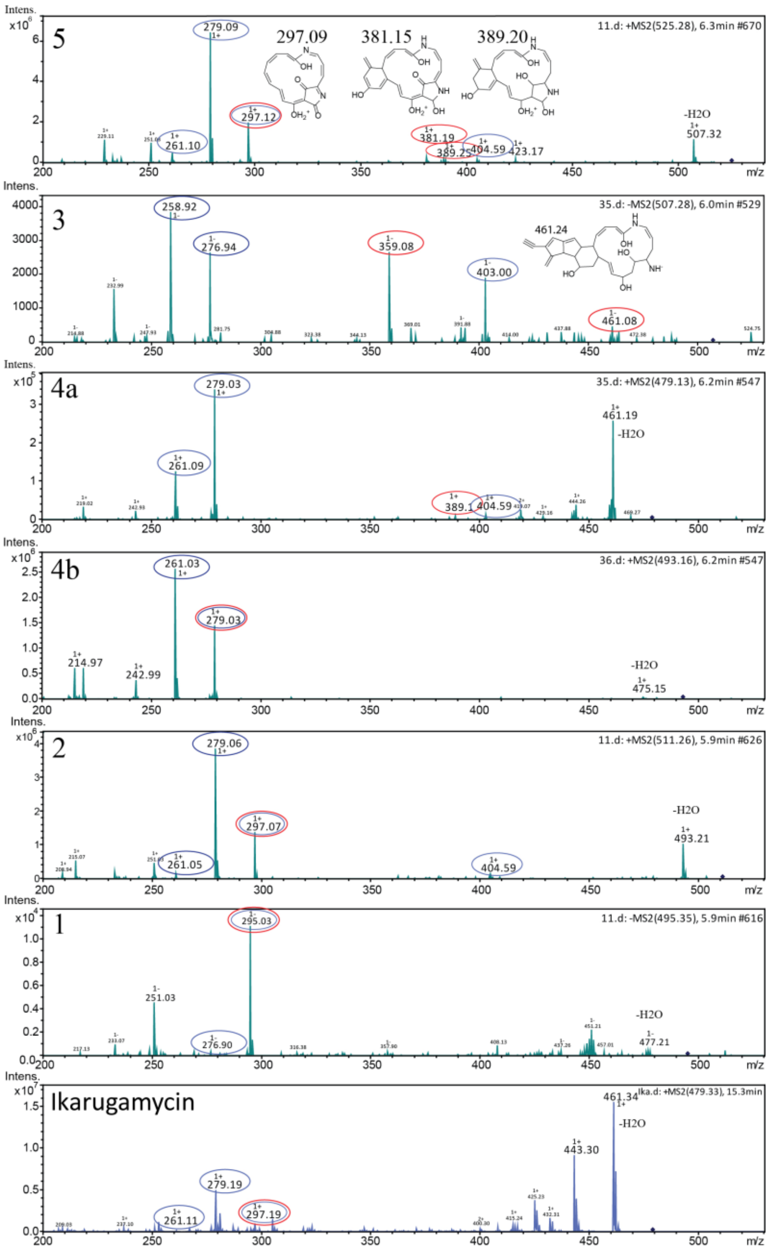

Figure 6S. MS/MS fragmentation pattern of compounds indetified in the extract of *S. albus* ATGSal2P2::Tn14 as PTMs and pure sample of ikarugamycin (Sigma-Aldrich, USA). Fragments marked with blue are common for all studied metabolites, including ikarugamycin. Fragments marked with red match the predicted fragmentation by CFM-ID software for particular compound [11]. Compound **5** fragmentation is similar to predicted for positive ion of frontalamide A. Compound **3** fragmentation is similar to predicted for negative ion of frontalamide B. Possible structures of fragments are shown (structures were generated by CFM-ID) Fragments with m/z of 261 $[M+H]^+$, 279 $[M+H]^+$ and 297 $[M+H]^+$ can be found in patterns of alteramide A and B deposited in GNPS Library (alteramide A ID: CCMSLIB00000077249; B: CCMSLIB00000077250) [12]. These fragments seem to be common for PTMs family. Mass-spec data was collected on LC-MS amaZon speed system (Bruker Daltonics, Germany).

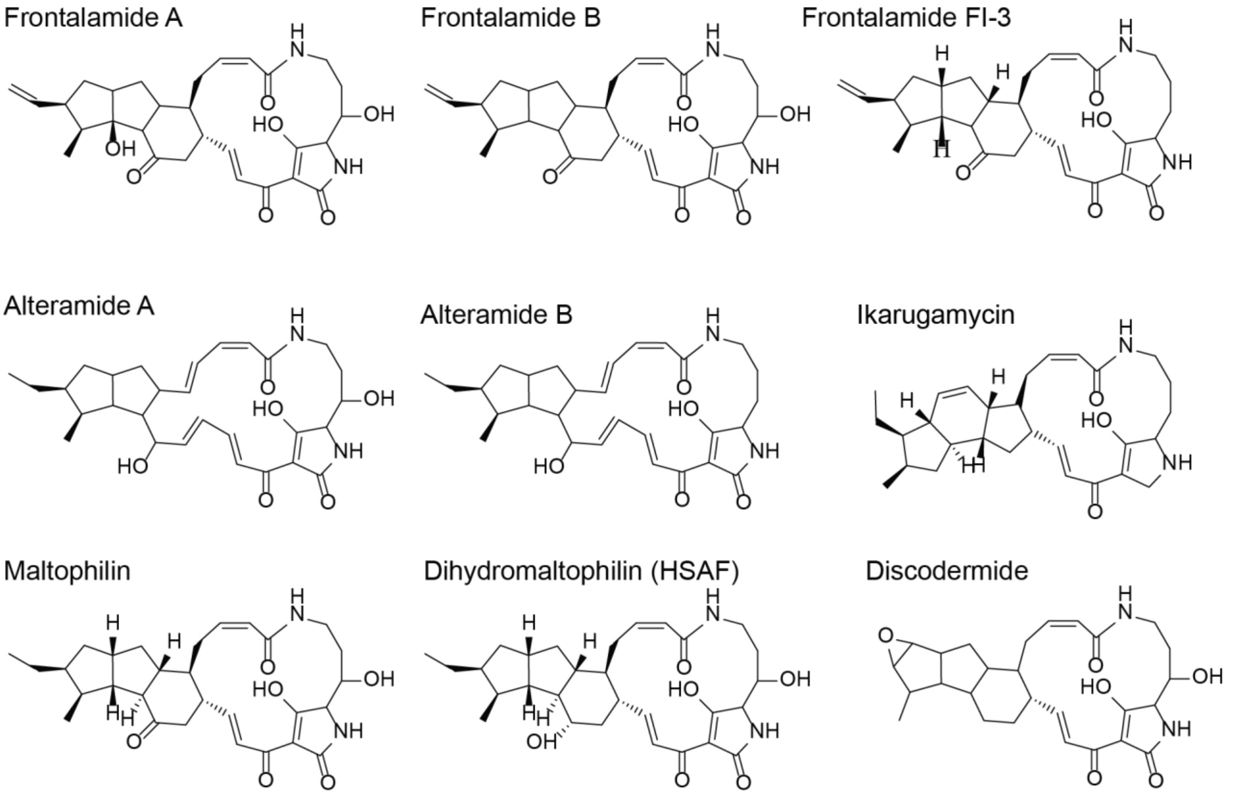

Figure 7S. Structures of several representatives of polycyclic tetramate mactolactams.

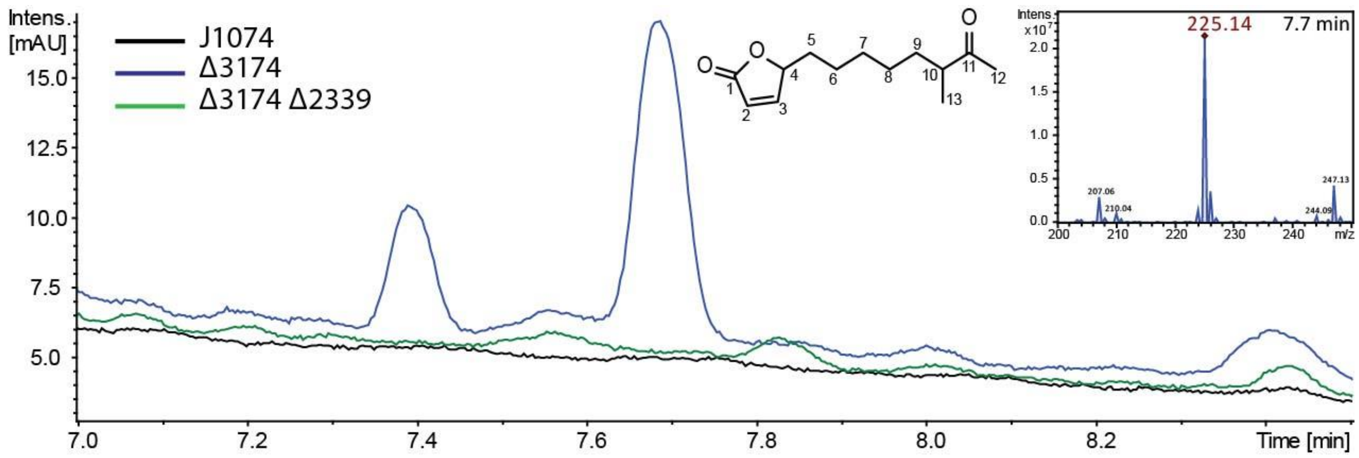

Figure 8S. Production of butenolide 4 by strains *S. albus* J1074 (black trace), *S. albus* Δ3174 (blue) and *S. albus* Δ3174Δ2339 (green). Chromatogram of 20 min gradient protocol (see Materials and Methods) at 320 nm is shown. Compound with retention time of 7.7 min was purified and its structure was proved with the NMR.

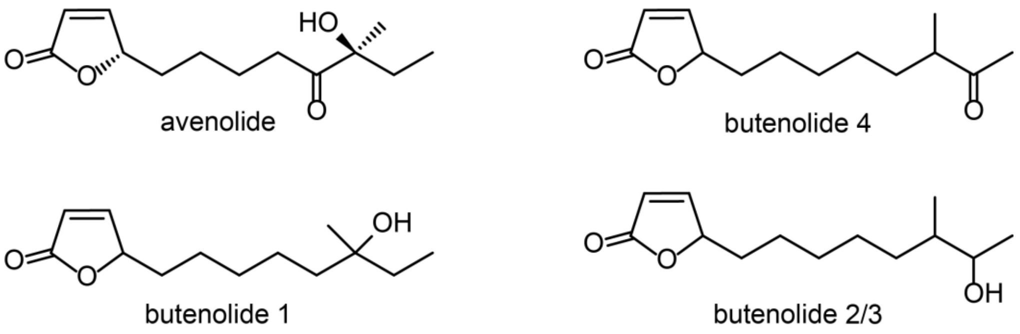

Figure 9S. Structures of avenolide from *S. avermitilis*, butenolide 4 from *S. albus*, and butenolides 1-4 from marine *Streptomyces* sp. SM8 and *S.* sp. B3497 [13,14].

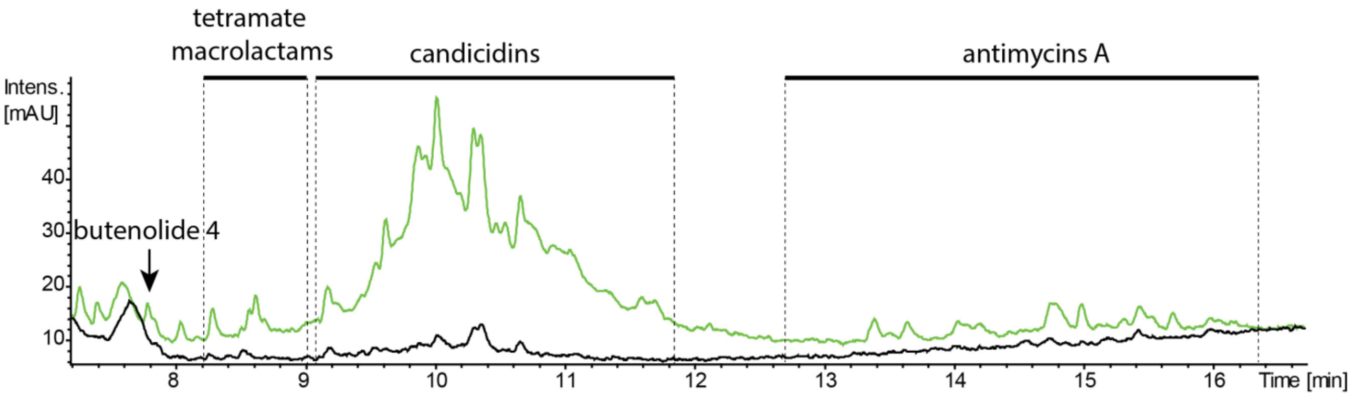

Figure 10S. LC-MS chromatogram (at 320 nm) of secondary metabolites extracts of *S. albus* J1074 (black) and *S. albus* J1074/pUWLH2339 (green) strains grown in NL19 medium. Samples were separated with the 20 min gradient protocol (see Materials and Methods). Identified compounds are highlighted.

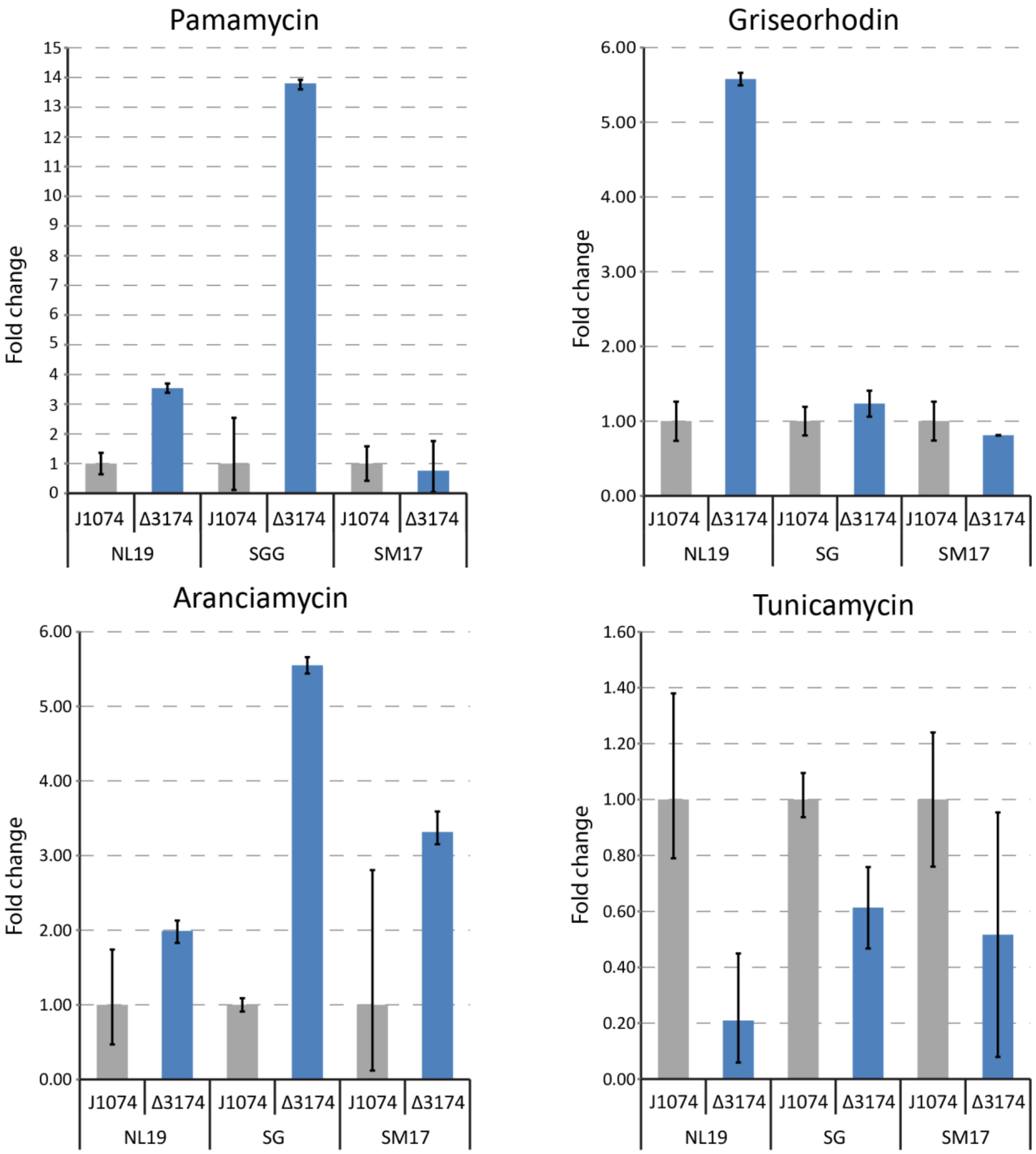

Figure 11S. Heterologous production of different secondary metabolites in *S. albus* J1074 and Δ3174.

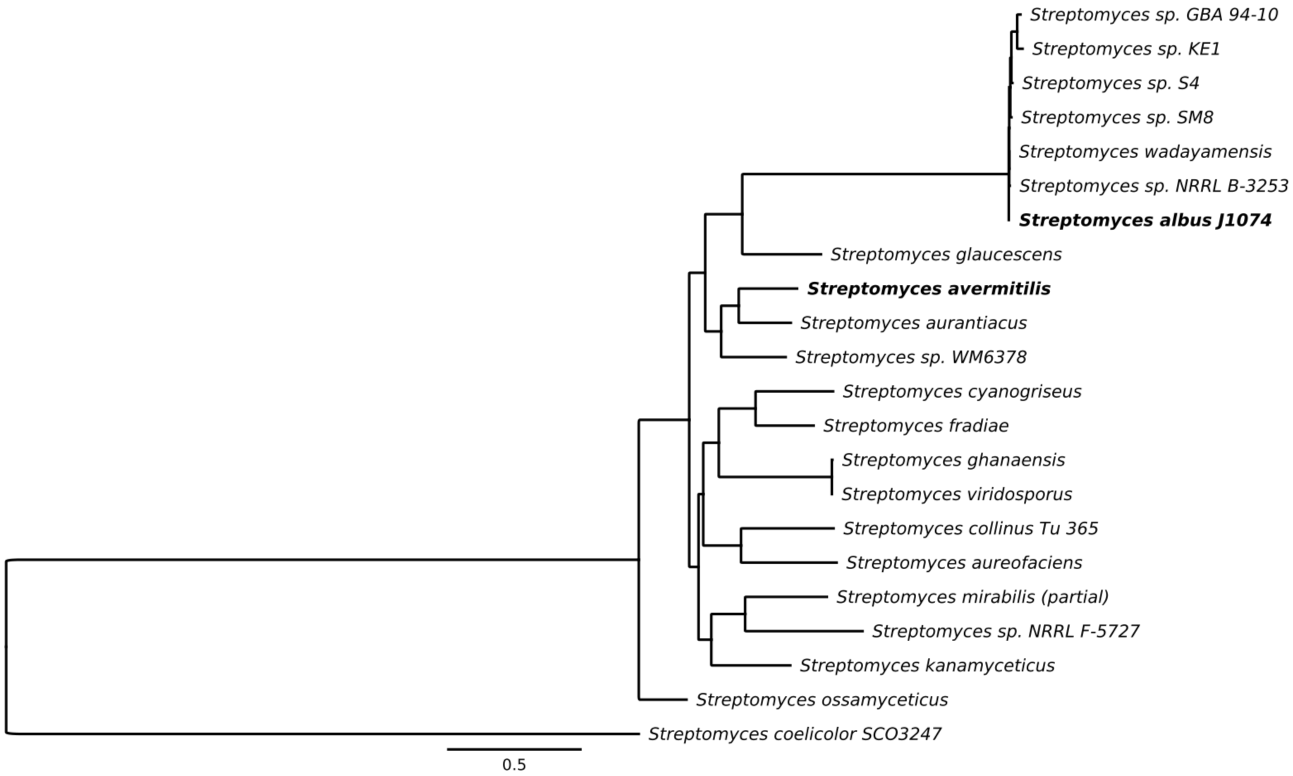

Figure 12S. Phylogeny of avenolide/butenolide biosynthesis acyl-CoA oxidase protein from different actinobacteria. Closest homologue from S. *coelicolor* was used as outgroup. The analysis and visualization was performed using Geneious software, version 8.1.7 (Biomatters Ltd, New Zealand).

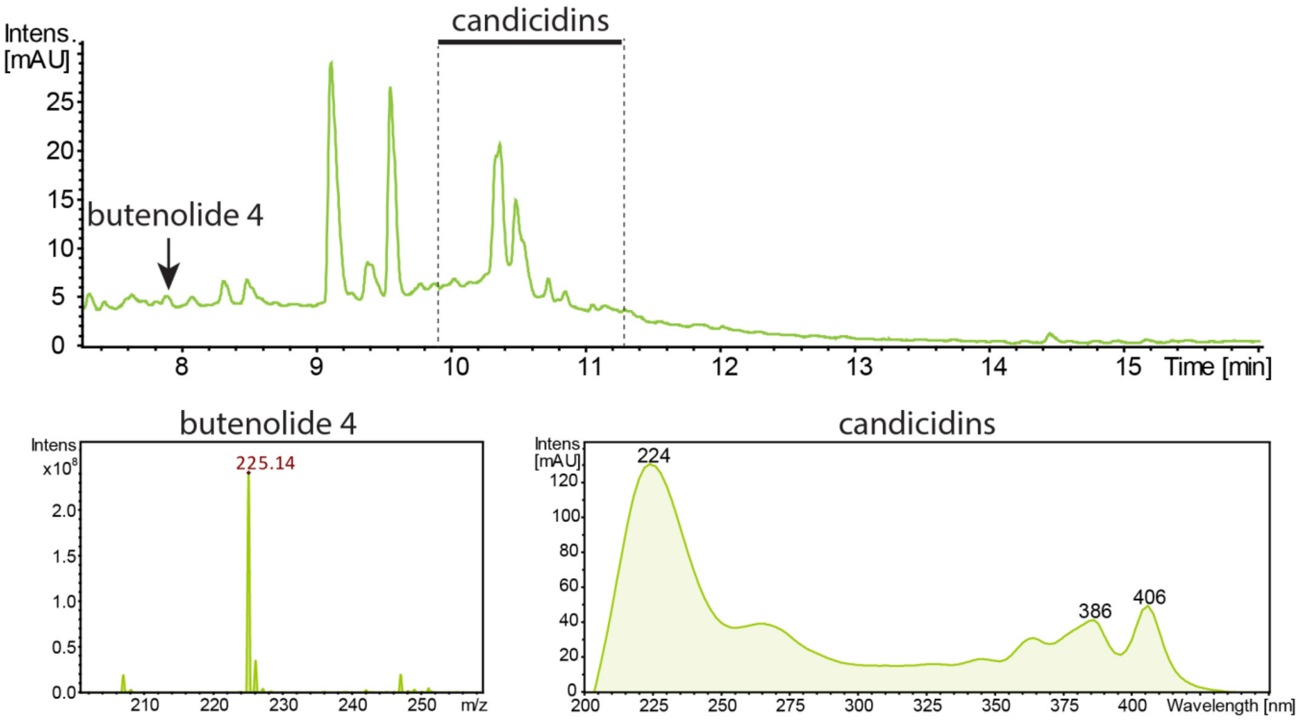

Figure 13S. LC-MS chromatogram (at 320 nm) of secondary metabolites extracts of *Streptomyces albidoflavus* NRRL B-1271 strain grown in NL19 medium. Sample was separated with the 20 min gradient protocol (see Materials and Methods). Identified compounds are highlighted.

References.

1 Chater, K. F. & Wilde, L. C. Restriction of a bacteriophage of *Streptomyces albus* G involving endonuclease *Sal*I. *J. Bacteriol.* **128**, 644-650 (1976).

2 Grant, S. G., Jessee, J., Bloom, F. R. & Hanahan, D. Differential plasmid rescue from transgenic mouse DNAs into *Escherichia coli* methylation-restriction mutants. *Proc. Natl. Acad. Sci. USA* **87**, 4645-4649 (1990).

3 Flett, F., Mersinias, V. & Smith, C. P. High efficiency intergeneric conjugal transfer of plasmid DNA from *Escherichia coli* to methyl DNA-restricting streptomycetes. *FEMS Microbiol. Lett.* **155**, 223-229 (1997).

4 Luzhetskyy, A. *et al.* Generation of novel landomycins M and O through targeted gene disruption. *ChemBioChem* **6**, 675-678, doi:10.1002/cbic.200400316 (2005).

5 Zaburannyi, N., Rabyk, M., Ostash, B., Fedorenko, V. & Luzhetskyy, A. Insights into naturally minimised *Streptomyces albus* J1074 genome. *BMC Genomics* **15**, doi:10.1186/1471-2164-15-97 (2014).

6 Luzhetskyy, A. *et al.* Cloning and heterologous expression of the aranciamycin biosynthetic gene cluster revealed a new flexible glycosyltransferase. *ChemBioChem* **8**, 599-602, doi:10.1002/cbic.200600529 (2007).

7 Li, A. & Piel, J. A gene cluster from a marine *Streptomyces* encoding the biosynthesis of the aromatic spiroketal polyketide griseorhodin A. *Chemistry & Biology* **9**, 1017-1026 (2002).

8 Wyszynski, F. J. *et al.* Biosynthesis of the tunicamycin antibiotics proceeds via unique exo-glycal intermediates. *Nat. Chem.* **4**, 539-546, doi:10.1038/nchem.1351 (2012).

9 Rebets, Y. *et al.* Insights into the pamamycin biosynthesis. *Angew. Chem. Int. Ed.* **54**, 2280-2284, doi:10.1002/anie.201408901 (2015).

10 Myronovskyi, M., Welle, E., Fedorenko, V. & Luzhetskyy, A. beta-Glucuronidase as a sensitive and versatile reporter in Actinomycetes. *Appl. Environ. Microbiol.* **77**, 5370-5383, doi:10.1128/Aem.00434-11 (2011).

11 Allen, F., Pon, A., Greiner, R. & Wishart, D. Computational Prediction of Electron Ionization Mass Spectra to Assist in GC/MS Compound Identification. *Anal. Chem.* **88**, 7689-7697, doi:10.1021/acs.analchem.6b01622 (2016).

12 Wang, M. *et al.* Sharing and community curation of mass spectrometry data with Global Natural Products Social Molecular Networking. *Nat. Biotechnol.* **34**, 828-837, doi:10.1038/nbt.3597 (2016).

13 Mukku, V. J., Speitling, M., Laatsch, H. & Helmke, E. New butenolides from two marine streptomycetes. *J. Nat. Prod.* **63**, 1570-1572 (2000).

14 Viegelmann, C. *et al.* Metabolomic profiling and genomic study of a marine sponge-associated *Streptomyces* sp. *Marine Drugs* **12**, 3323-3351, doi:10.3390/md12063323 (2014).
